# The Plant Pathogenic Bacterium *Streptomyces scabies* Degrades the Aromatic Components of Potato Periderm via the β-Ketoadipate Pathway

**DOI:** 10.3389/fmicb.2019.02795

**Published:** 2019-12-04

**Authors:** Mario Khalil, Sylvain Lerat, Nathalie Beaudoin, Carole Beaulieu

**Affiliations:** ^1^Département de Biologie, Centre SÈVE, Université de Sherbrooke, Sherbrooke, QC, Canada; ^2^Department of Microbiology, Faculty of Science, Ain Shams University, Cairo, Egypt

**Keywords:** common scab, hydroxycinnamates, *trans*-ferulic acid, *Streptomyces scabiei*, suberin

## Abstract

The outer potato periderm layer consists of dead suberized cells. Suberin, a protective biopolymer, is made of a polyaliphatic portion covalently linked to polyaromatic moieties. Evidence accumulates that *Streptomyces scabies*, the main causal agent of potato common scab, can degrade the suberin aliphatic part but its ability to degrade the aromatic portion has not been documented. This polyaromatic portion is mainly composed of cinnamic acids. In this study, two cinnamates (*trans*-ferulic or *p*-coumaric acids) were added to the culture medium of *S. scabies* strains EF-35 and 87.22. HPLC quantification revealed that both strains efficiently utilized these compounds. A proteomic study coupled with gene expression analysis led to the identification of putative catabolic pathways for cinnamates. Catabolism of both compounds appeared to occur via the β-ketoadipate pathway. Gene SCAB_15301, encoding for a putative vanillate monooxygenase, was partly deleted from *S. scabies* strain 87.22 genome. The mutant retained its ability to catabolize *trans*-ferulic acid into vanillate but lost its ability to further degrade the latter compound. When the wild-type mutant and complemented strains were grown in the presence of suberin-enriched potato periderm, accumulation of vanillic acid was observed only in the mutant culture medium. This work presents evidence that *S. scabies* can degrade not only the aliphatic part of suberin but also the constituents of suberin aromatic portion. This may provide ecological and pathological advantages to *S. scabies* as a saprophyte and pathogen.

## Introduction

*Streptomyces scabies* is the main causal agent of potato common scab, a disease which reduces both crop value and tuber marketability. This disease is characterized by dark colored lesions covering the tubers surface. The pathogenicity of *S. scabies* is mainly caused by the production of toxins known as thaxtomins, which induce cell death in plant cells and tissues (Goyer et al., 1998; Healy et al., 2000; Duval et al., 2005).

The potato skin contains high levels of suberin, a biopolymer playing a protective role against pathogen entry in potato tuber tissues. Suberin is a complex polymer consisting of two covalently linked portions (see model proposed by Bernards, 2002). The polyaliphatic portion located between the plasma membrane and the primary cell wall consists of esterified long-chain fatty acids. Evidence accumulates that *S. scabies* produces esterases degrading this aliphatic part (Beaulieu et al., 2016). A secretome analysis of *S. scabies* cultures grown in the presence of suberin was performed to identify the enzymes potentially involved in the degradation of suberin (Komeil et al., 2014). Glycosyl hydrolases were the most abundant proteins in the supernatant of suberin-containing medium, in addition to several enzymes involved in lipid metabolism (Komeil et al., 2014).

The polyaromatic portion of suberin is a lignin-like structure embedded in the primary cell wall. It is mainly composed of polyhydroxycinnamates (Bernards et al., 1995; Bernards and Lewis, 1998), where *trans*-ferulic and *p*-coumaric acids figure among the main hydroxycinnamates forming the aromatic moiety of suberin (Riley and Kolattukudy, 1975). Previous work has shown the importance of *trans*-ferulic acid in keeping the integrity of both suberin and periderm wax (Serra et al., 2010), therefore preventing the pathogen entry.

Suberin degradation, especially of the aromatic part, has been mostly investigated in fungi. As an example, *Aspergillus nidulans* was reported to degrade the aromatic part of suberin by monitoring the degradation products when *A. nidulans* was grown on suberized cell walls (Martins et al., 2014). The microbial degradation of suberin is a process that has been poorly characterized. However, there are several *Streptomyces* species that have been reported to degrade aromatic compounds. *Streptomyces* sp. strain ERI-CPDA-1, isolated from oil contaminated soil, was able to degrade petroleum and polycyclic aromatic hydrocarbons (PAHs). The degradation products detected were benzaldehyde, catechol, phenylacetic acid and protocatechuic acid (Balachandran et al., 2012). *Streptomyces setonii* strain ATCC 39116, a thermophilic soil actinomycete, was also shown to be able to degrade single aromatic compounds, including phenol and benzoate, through the ortho-cleavage pathway (Park and Kim, 2003).

A previous study showed that in the presence of suberin, *S. scabies* secreted the extracellular protein C9Z2P6, a putative 3-oxo-5,6-dehydrosuberyl-CoA semialdehyde dehydrogenase, which might be involved in the degradation of aromatic compounds. Moreover, the gene encoding this protein was overexpressed in presence of suberin (Komeil et al., 2014), suggesting that *S. scabies* has the ability to degrade the aromatic part of suberin.

In the present study, the ability of *S. scabies* to degrade the main aromatic constituents of the potato suberin, *trans*-ferulic acid and *p*-coumaric acid, is investigated. A hypothetical degradation pathway of *trans*-ferulic and *p*-coumaric acids in *S. scabies* 87.22 is proposed.

## Materials and Methods

### Culture Conditions

Strains and plasmids used in this study are listed in Table 1. Inocula of *S. scabies* strains were prepared by inoculating approximately 10^8^ spores in 50 mL of yeast malt extract (4 g L^–1^ glucose, 4 g L^–1^ yeast extract, and 10 g L^–1^ malt extract). The bacteria were then incubated at 30°C with shaking (250 rpm) for 48 h followed by bacterial cells recovery using centrifugation (3,500 × *g*) for 10 min. The collected pellets were resuspended in 5 volumes of saline solution (NaCl 0.85%) and the resulting bacterial suspensions were used as inocula in further experiments. These bacterial inocula (100 μL) were added to 50 mL of the control medium (CM) composed of 0.5 g L^–1^ l-asparagine, 0.5 g L^–1^ K_2_HPO_4_, 0.2 g L^–1^ MgSO_4_⋅7H_2_O, 10 mg L^–1^ FeSO_4_⋅7H_2_O, and 0.05% (w/v) casein hydrolysate. This medium could be supplemented with *trans*-ferulic acid (60 μM), *p*-coumaric acid (50 μM), vanillic acid (60 μM), protocatechuate (60 μM), suberin 0.1% (w/v) or a combination of both ferulic and vanillic acids. Suberin was purified from potato tubers according to Kolattukudy and Agrawal (1974) with modifications proposed by Komeil et al. (2013). Culture media were adjusted to pH 7.0 and incubated with shaking (250 rpm) at 30°C. *Escherichia coli* strains were grown on LB agar plates at 37°C. When necessary, spectinomycin (100 μg mL^–1^ final concentration) was added to the culture medium.

**TABLE 1:** Bacterial strains and plasmids used in this study.

**Strain or plasmid**	**Description**	**References**
**Strains**		
*Streptomyces scabies* EF-35	Wild strain	Faucher et al., 1992
*S. scabies* 87.22	Wild strain	Loria et al., 1995
*S. scabies* ΔSCAB_15301	*S. scabies* 87.22 carrying a non-functional SCAB_15301 gene	This work
*S. scabies* com15301	ΔSCAB_15301 complemented with a functional SCAB_15301 gene carried by pSET15301	This work
*Escherichia coli* NEB 5-alpha	High-efficiency competent strain	New England Biolabs
*E. coli* ET12567	Non-methylating conjugation strain	MacNeil et al., 1992
**Plasmids**		
pUZ8002	Non-transmissible RP4 derivative plasmid	Kieser et al., 2000
pCRISPomyces-2	Plasmid for targeted genome editing in *Streptomyces* species	Cobb et al., 2015
pCRISPo-proto15301	The pCRISPomyces-2 plasmid containing a SCAB_15301-specific sequence that the Cas9 system can target	This study
pCRISPo-15301-full	The pCRISPo-proto15301 plasmid with a partially deleted SCAB_15301 gene inserted in a unique *Xba*I site	This study
pSET152m	Modified pSET152 vector in which the apramycin resistance gene *aac(3)IV* is replaced with the spectinomycin resistance gene *aadA*	Lu et al., 2007
pSET15301	pSET152m derivative carrying SCAB_15301 gene	This study

### Quantification of Cinnamic Acids in *Streptomyces scabies* Culture Broths

Amounts of *trans*-ferulic, *p*-coumaric and vanillic acids in culture broths were quantified by high performance liquid chromatography (HPLC). These cinnamic acids were detected using an Agilent 1260 Infinity high-performance liquid chromatograph equipped with a reverse-phase C18 column with fully porous particles of 3.5 μm size (Zorbax SB-C18, 4.6 × 150 mm, Agilent). Cinnamic acids were eluted using a 25 to 75% acetonitrile linear gradient for 10 min at a flow rate of 1 mL min^–1^ and monitored at 310 nm (*trans*-ferulic and *p*-coumaric acids) or 254 nm (vanillic acid). Standard curves were established for each compound analyzed using dilutions of known quantity of the compounds.

### Proteomics Analysis

*Streptomyces scabies* strain 87.22 was grown in CM for 4 days. Six and 12 h later, *trans*-ferulic acid (60 μM) was then added or not to the culture medium and the culture was incubated for an additional 6 h. Bacterial cells were then collected by centrifugation at 3,500 × *g* for 10 min and were washed with phosphate buffered saline (PBS). PBS was removed by centrifugation and the intracellular proteins of collected cells were extracted by adding PBS and freezing the cells in ethanol kept at –80°C. The cells were thawed for 10 min at room temperature. This freeze-thaw lysis was repeated twice, and the suspension was sonicated for 10 s. The suspension was centrifuged at 4°C for 10 min (3,500 × *g*) and supernatant containing the proteins was kept at 4°C. Intracellular proteins were subjected to sodium dodecyl sulfate-polyacrylamide gel electrophoresis [10% (w/v) SDS-PAGE] and in-gel protein digestion were carried out according to Komeil et al. (2013). Mass spectrometry was conducted at the Proteomics Platform of the Quebec Genomics Center (Quebec City, Canada) using a hybrid quadrupole time-of-flight (QqTOF) (TripleTOF 5600 plus, SCIEX) coupled to a capillary HPLC for peptide separation via a nanospray ionization source. All MS/MS spectra were then interpreted using Mascot (Matrix Science, London, United Kingdom) to search *S. scabies* strain 87.22 Uniref100 database to provide statistically validated matches between observed spectra and identified peptides and list of proteins. The results were then uploaded to the scaffold software program (version Scaffold 4.8.8, Proteome Software, Portland, OR, United States) and a filter was set with a 95% minimum protein ID probability with a minimum number of two unique peptides, in which the cut-offs for peptide thresholds were set to 1.0% false discovery rate (FDR). Protein function was predicted using the UniProt, NCBI, KEGG, and COG databases. The normalized spectral count (NSpC) of proteins was obtained by dividing the number of spectra (SpC) for a protein by the molecular weight (MW) of the corresponding protein (Neilson et al., 2011). The experiment was done in duplicate.

### qRT-PCR

The expression of the potential cinnamic acid catabolic genes was determined in the presence of ferulate or coumarate as follows. *S. scabies* strain 87.22 was grown in CM for 4 days and then *trans*-ferulic or *p*-coumaric acid was added or not to the culture medium. Three hours later, 10 mL of each culture medium was mixed with 2 mL of stop solution (ethanol/acidic phenol, 95:5, [v/v]) to prevent RNA degradation (Joshi et al., 2007). Bacterial cell pellets were recovered by centrifugation at 4°C for 10 min at 3,500 × *g* and stored at –80°C until further use. RNA was extracted from cells using the RNeasy Mini kit (Qiagen) (Lerat et al., 2010). Two μg of total isolated RNA were then reverse transcribed to cDNA using the Maxima First strand cDNA synthesis kit (ThermoFisher Scientific) according to the manufacturer’s instructions. Diluted cDNA (10×) was used to perform quantitative real-time reverse-transcription polymerase chain reaction (qRT-PCR) using Mx3000P qPCR system (Agilent Technologies) with the BrightGreen 2x qPCR MasterMix-Low ROX. The q-RT PCR conditions were 95°C for 10 min followed by 35 cycles at 95°C for 15 s and 60°C for 30 s. Relative expression levels were determined by using the comparative C_T_ values according to Pfaffl (2001) with *gyrA* gene as a reference gene. Primers used in this assay are listed in Table 2.

**TABLE 2:** Primers used in the gene expression assay.

**Gene assignation**	**Predicted function of the corresponding protein**	**Primer sets (5′–3′)**
SCAB_13631	Protocatechuate-3,4-dioxygenase beta subunit	For:CACATCCACTTCTCGCTCTT
		Rev:ACTGGATGATCGGGTCGTA
SCAB_13661	3-oxoadipate enol-lactone hydrolase	For:CGCTTCCAGGACTTCATCTC
		Rev:CATGGCCAGTTCGTCGTAG
SCAB_15301	Vanillate monooxygenase alpha subunit	For:CAACCACACGGTCGTCAT
		Rev:GATGTTGATGCTCAGCTCCT
SCAB_15321	Iron-sulfur oxidoreductase beta subunit	For:CTACGTGCACACGGAGTT
		Rev:GGTTCCAGGGCGAAGTT
SCAB_15331	IclR-family transcriptional regulator	For:GAGCATCCCGCACTGAC
		Rev:CGAGCGTGAGCAGGAAG
SCAB_15591	Feruloyl-CoA hydratase	For:CACCCTGTCGCTGTTCAT
		Rev:CCTTGTTGAGGCCGTAGTT
SCAB_15601	Feruloyl-CoA synthetase	For:GACGACACCTGCATCATCA
		Rev:GTTGACGGCGTTCCAGAT
SCAB_2141	2,3-dihydroxy-2,3-dihydro-phenylpropionate dehydrogenase	For:GACGAGATCTTCGCGATCAA
		Rev:GCGTTGGACAGGGTCAT
SCAB_45751	Gyrase A (*gyrA*)	For:GCCATCAACCTCCGTGAAA
		Rev:CGGATCGATTGTGCCTTCTT
SCAB_6611	3-oxoadipate CoA-transferase subunit	For:CTACGTGATGATGACGCTCTT
		Rev:ACCGTGGTCGGTGTAGA
SCAB_6621	3-oxoadipate CoA-transferase subunit A	For:CTCACCATCGTCTCCAACAA
		Rev:AAGGAGCAGAGCACCTTG

### Deletion of the Putative Vanillate Monooxygenase Gene SCAB_15301 From *Streptomyces scabies* 87.22 Genome

*Streptomyces scabies* strain 87.22 carrying a non-functional SCAB_15301 gene was constructed using the CRISPR-Cas9 system. The procedure of Wang et al. (2016), specifically developed for *Streptomyces* species, was followed with plasmid pCRISPomyces-2 (Addgene) to carry the construction.

Briefly, a short DNA sequence, unique to SCAB_15301 in the *S. scabies* genome (protospacer: CCGGTCGACCTCGCGTAGAA), was introduced into pCRISPomyces-2, generating plasmid pCRISPo-proto15301. A partially deleted version of gene SCAB_15301 (generating a stop codon) was then created, using the Gibson Assembly Master Mix (New England Biolabs). Pairs of primers used to create the homology arms required for the Gibson assembly were 5′-GTTGCCGCCGGGCGTTTTTTATCCTTCCTGACCTGCGAT AAC-3′ + 5′-GATCCGCCCCTCGATGGCGGGCACTCCGCGT CGTCCATGT-3′ and 5′- ACGCGGAGTGCCCGCCATCGAGG GGCGGATCACCCGCTG-3′ + 5′- GCCTTTTTACGGTTCCT GGCCTGTTCCAGGGCGAAGTTGTT -3′-). The resulting DNA construct was inserted in pCRISPo-proto15301 and the obtained plasmid was named pCRISPo-15301-full. This region serves as repair template to the recombination process that follows the disruption of target DNA by the CRISPR-Cas9 system.

pCRISPo-15301-full was introduced in the *E. coli* conjugation strain ET12567-pUZ8002. Conjugation between *S. scabies* 87.22 and *E. coli* ET12567- pUZ8002 carrying plasmid pCRISPo-15301-full was performed as described in Kieser et al. (2000). The insertion of non-functional SCAB_15301 gene in the genome of exconjugants was confirmed by DNA sequencing using primers annealing slightly upstream and downstream of the editing template sequence (5′-CTTCTGCGAGGAAACCAACA-3′ and 5′-CAGCATCGGCAGGATGG-3′). Finally, the plasmid carrying the pCRISPomyces system was cleared from the *S. scabies* recombinant strain after three serial cultures in ISP-2 medium (Wang et al., 2016).

### Complementation of ΔSCAB_15301 Deletion Mutant

Genomic DNA was obtained from *S. scabies* strain 87.22 using the salting-out procedure (Kieser et al., 2000). SCAB_15301 along with the upstream region (1799 bp) were amplified from the wild type strain using two primers to which the restriction sequences of the digestion enzymes were added (5′-CGGAATTCCGTCTCCTCCTCGGTCCTCACCC-3′ and 5′-GCTCTAGAGCTCACAGGACCTTCTCCACCGG-3′). PCR reactions were carried out in a final volume of 50 μL containing 1 μL (233 ng μL^–1^) of template DNA, 2.5 μL (10 μM) of each primer, 10 μL of 10 × buffer, 1 μL of 10 mM dNTPs, 0.5 μL of Q5^®^ high-fidelity DNA polymerase, 10 μL of Q5 high GC enhancer. The PCR cycling conditions were as follows: an initial pre-denaturing step at 98°C for 30 s, 35 cycles at 98°C for 10 s, 72°C for 1 min and a final extension step at 72°C for 2 min using Bio-Rad T100 thermal Cycler.

Amplicons of the putative vanillate monooxygenase gene SCAB_15301 and integrative vector pSET152m (Lu et al., 2007) were both digested with *Eco*RI and *Xba*I at 37°C for 2 h. SCAB_15301 gene was ligated into the digested plasmid using DNA ligase at 16°C for 2 h. The resulting plasmid pSET15301 was transformed to *E. coli* ET12567-pUZ8002. The transformation was done as described by Kieser et al. (2000). The conjugation between *E. coli* ET12567-pUZ8002-pSET15301 and *S. scabies* ΔSCAB_15301 was carried out as proposed by Kieser et al. (2000). The complementation was confirmed by sequencing the inserted gene SCAB_15301 using the same primers used for SCAB_15301 amplification. Sequencing was carried out at Genome Sequencing and Genotyping Platform (Quebec City, Canada) using Sanger technique.

## Results

### Utilization of Cinnamic Acids by *Streptomyces scabie*s

**FIGURE 1:**
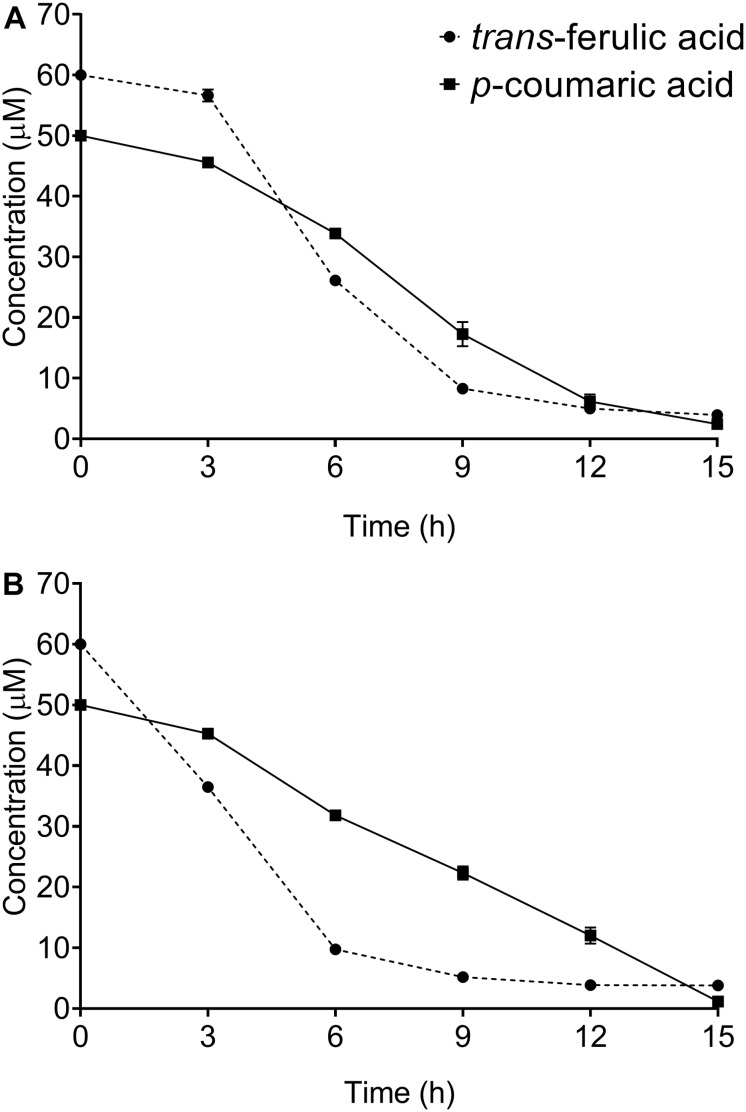
Kinetics of *trans*-ferulic and *p*-coumaric acids utilization in *Streptomyces scabies* strains EF-35 **(A)** and 87.22 **(B)**. Data shown are the mean of three replicates (±SD).

A time course study of both *trans*-ferulic and *p*-coumaric acids depletion was carried out by sampling the *S. scabies* culture at 3-h intervals for 15 h. Both *S. scabies* strains EF-35 and 87.22 gradually utilized these compounds. After 15 h of incubation, most of *trans*-ferulic and *p*-coumaric acids were consumed (Figures 1A,B). During the first hours of incubation, both tested strains showed higher degradation affinity toward *trans*-ferulic acid when compared to *p*-coumaric acid, and especially *S. scabies* 87.22 which consumed about 84% of the added *trans*-ferulic acid after 6 h of inoculation (Figure 1B).

**FIGURE 2:**
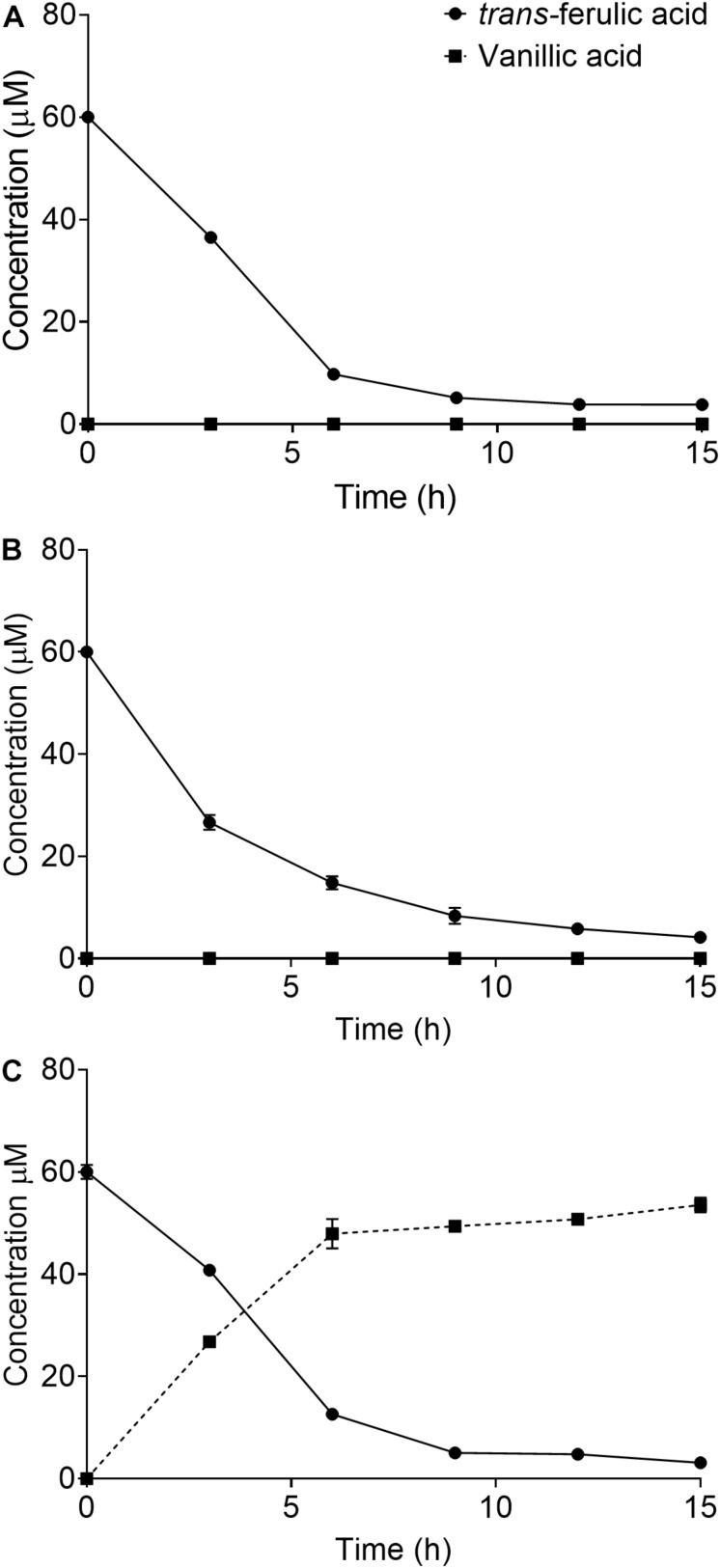
Utilization of ferulic acid by *S. scabies*: **(A)** *S. scabies* 87.22 utilized *trans*-ferulic acid but did not accumulate vanillate. **(B)** *S. scabies* com15301 utilized *trans*-ferulic acid without vanillate accumulation. **(C)** ΔSCAB_15301 converted over 80% of the initial amount of *trans*-ferulic acid into vanillate within 9 h. Data shown are the mean of three replicates (±SD).

**FIGURE 3:**
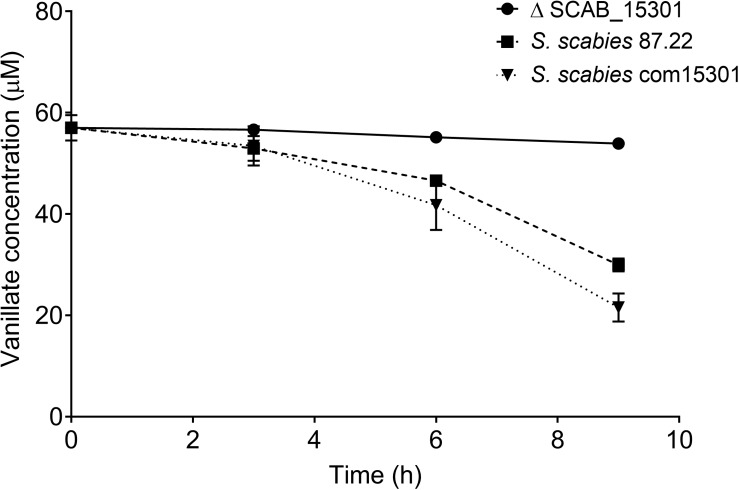
Catabolism of vanillate in *S. scabies* strains 87.22, ΔSCAB_15301 and com15301. Data shown are the mean of three replicates (±SD).

SCAB_15301 has been identified in the *S. scabies* 87.22 genome [using basic local alignment search tool (BLAST)] as a gene encoding a vanillate monooxygenase (Supplementary Figure S1), which is responsible for bioconversion of *trans*-ferulic acid into protocatechuate. To confirm the predicted function of this gene, SCAB_15301 was deleted from *S. scabies* 87.22 genome using CRISPR/Cas9-based method. The CRISPR/Cas9-based method has been shown to be effective in *Streptomyces* genome editing (Wang et al., 2016). However, the success rate in *S. scabies* using the CRISPR/Cas9-based system was very low as only one exconjugant was obtained and all efforts to obtain other mutants failed. Nonetheless, depletion of ferulic acid by the deletion mutant *S. scabies* ΔSCAB_15301 was observed. This mutant was able to convert over 80% of the initial amount of ferulic acid into vanillate within 9 h (Figure 2C). This contrasted with both the wild and complemented strains, where vanillate did not accumulate into the culture medium (Figures 2A,B). Moreover, when vanillic acid was added to the culture medium of strains 87.22, ΔSCAB_15301 and *S. scabies* com15301, catabolism of vanillate was observed in strain 87.22 and the complemented strain but not in ΔSCAB_15301 (Figure 3), indicating that SCAB_15301 effectively codes for a putative vanillate monooxygenase.

### Effect of *Trans*-Ferulic Acid on *Streptomyces scabies* 87.22 Proteome

Intracellular proteins produced by *S. scabies* 87.22 during growth in CM supplemented or not with *trans*-ferulic acid are listed in Supplementary Table S1. Sixty-nine proteins were found exclusively in the presence of *trans*-ferulic acid. These proteins were categorized according to their functional groups (Table 3). Among them, seven proteins were identified to be potentially involved in the degradation of aromatic compounds (Table 3) and five were included in the stress mechanism functional group. Only two proteins were detected exclusively in CM without *trans*-ferulic acid; a transmembrane efflux protein (C9Z0L4) with NSpC of 0.03 and a membrane protein with unknown function with NSpC of 0.04.

**TABLE 3:** Proteins detected only in *Streptomyces scabies* 87.22 proteome in control medium supplemented with ferulic acid.

**Uniprot accession #**	**Corresponding gene in *S. scabies* 87.22**	**Predicted function**	**Abundance (NSpC)**
**Degradation of aromatic compounds**			
C9ZBH8	SCAB_15301	Vanillate monooxygenase	0.10
C9YX73	SCAB_6611	3-oxoadipate CoA-transferase subunit	0.12
C9YX74	SCAB_6621	3-oxoadipate CoA-transferase subunit A	0.16
C9ZBI0	SCAB_15321	Iron-sulfur oxidoreductase beta subunit	0.08
C9ZBK6	SCAB_15591	Enoyl-CoA hydratase	0.18
C9ZBK7	SCAB_15601	Feruloyl-CoA synthetase	0.05
C9Z892	SCAB_13631	Protocatechuate 3,4-dioxygenase beta subunit	0.10
**Carbohydrate transport and metabolism**			
C9Z6Y3	SCAB_28241	Pyruvate kinase	0.11
C9ZGJ3	SCAB_18361	Malate synthase	0.11
C9Z376	SCAB_86561	Alpha-L-rhamnosidase	0.02
C9Z510	SCAB_11461	Succinate dehydrogenase	0.11
C9ZF59	SCAB_33461	Polyphosphate glucokinase	0.07
C9YZG5	SCAB_54911	UDP-glucose 6-dehydrogenase	0.04
**Stress mechanism**			
C9Z1S1	SCAB_9141	Betaine aldehyde dehydrogenase	0.34
C9Z9F8	SCAB_76571	Uracil-DNA glycosylase	0.12
C9YZC4	SCAB_39071	Hydroxymethylbilane synthase	0.05
C9ZDL6	SCAB_47371	Type II toxin-antitoxin	0.18
C9YX55	SCAB_83581	Aspartate ammonia-lyase	0.04
**Lipid metabolism**			
C9ZBK6	SCAB_15591	MaoC family dehydratase	0.18
C9ZBK7	SCAB_15601	Fatty-acyl-CoA synthase	0.05
C9Z9V3	SCAB_0081	Hydrolase	0.05
C9YVX2	SCAB_20961	Fatty acid oxidation complex	0.11
**Transcriptional, translational, ribosomal structure, and biogenesis**			
C9Z4I3	SCAB_74011	Phenylalanine tRNA ligase	0.06
C9Z626	SCAB_74721	Exonuclease	0.15
C9Z1C5	SCAB_72001	GTPase Der	0.02
C9Z0Y8	SCAB_55691	Peptide chain release factor 2	0.07
C9ZAL3	SCAB_45881	Methyltransferase G	0.07
C9Z669	SCAB_75161	Transcription antitermination protein	0.12
C9Z0W5	SCAB_39991	50S ribosomal protein L31 type B	0.20
C9YUZ3	SCAB_36381	Transcriptional regulator	0.05
C9Z1E8	SCAB_72241	Transcriptional repressor	0.07
C9Z633	SCAB_74791	Elongation factor	0.02
**Signal transduction mechanism**			
C9Z568	SCAB_26741	Histidine kinase	0.01
C9ZGN5	SCAB_33821	Phosphorelay signal transduction pilus	0.03
**Nucleotide transport and metabolism**			
C9ZF48	SCAB_33351	Nucleotide modification protein	0.08
C9Z0P9	SCAB_39321	Phosphatase	0.06
C9ZGK9	SCAB_18521	Uricase	0.05
**Cell wall/membrane/envelope biogenesis**			
C9YVQ7	SCAB_6101	Conserved protein (MreB, spore wall)	0.07
**Coenzyme transport and metabolism**			
C9Z245	SCAB_25301	Riboflavin biosynthesis protein	0.05
C9Z8W6	SCAB_45281	Guanylyltransferase	0.06
C9Z1Y6	SCAB_9791	Precorrin-8X methylmutase	0.08
**Amino acid transport and metabolism**			
C9Z5A1	SCAB_27071	Acetolactate synthase small subunit	0.10
C9ZGY7	SCAB_49631	Sulfurtransferase	0.09
C9Z6A7	SCAB_88411	Dihydroxy-acid dehydratase	0.03
C9YZD9	proC	Pyrroline-5-carboxylate reductase	0.06
C9ZBN4	SCAB_15871	Oxidoreductase	0.05
C9Z8G8	SCAB_28961	Homoserine dehydrogenase	0.04
C9ZBK8	SCAB_15611	Amidohydrolase	0.12
**Transport, secretion, and efflux**			
C9ZC07	SCAB_46421	ABC transporter (cytochrome bd)	0.01
C9YX17	SCAB_83191	Transport system integral protein	0.05
C9ZGM4	SCAB_18691	Cation transport protein (Mg)	0.07
C9ZAS1	SCAB_61981	Metal-binding lipoprotein (Zn)	0.05
**General function prediction only**			
C9ZBK9	SCAB_15621	Dehydrogenase/reductase	0.27
C9ZCN6	SCAB_78601	Ligase	0.03
C9ZE91	SCAB_79211	RarE (Conservon) homolog	0.04
C9Z4V2	SCAB_87941	Hydrolase	0.09
C9YU23	SCAB_81961	Ligase and argininosuccinate lyase	0.06
C9Z2U3	SCAB_72631	Aldehyde dehydrogenase	0.04
C9Z7E5	SCAB_59891	Serine/threonine-protein phosphatase	0.04
C9ZAA1	SCAB_29911	Putative methyltransferase	0.07
**Unknown function**			
C9Z8Y5	SCAB_60551		0.15
C9ZH42	SCAB_50211		0.11
C9Z8 × 7	SCAB_60471		0.13
C9Z0T5	SCAB_39691		0.03
C9ZDU8	SCAB_48211		0.06
C9YZT7	SCAB_71141		0.06
C9YTB7	SCAB_35231		0.11
C9YZA8	SCAB_38901		0.05
C9Z0S1	SCAB_39541		0.33

### Effects of Hydroxycinnamates on the Expression of the Putative Aromatic Compounds Degradation Genes

**FIGURE 4:**
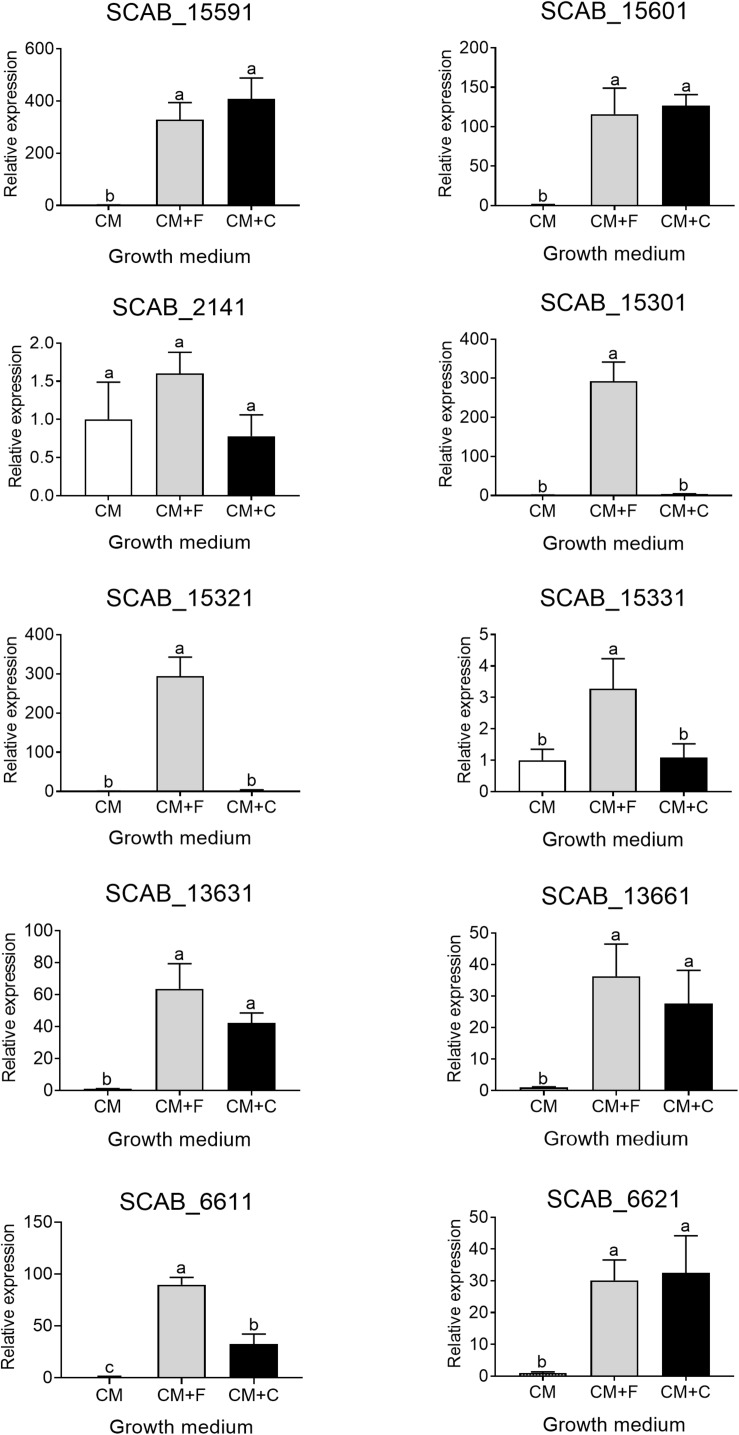
Relative expression levels (+SD) of targeted genes involved in *trans*-ferulic and *p*-coumaric acids degradation from *S. scabies* 87.22 grown in control medium (CM) alone, CM supplemented with *trans*-ferulic acid (CM+F, gray bars) and CM supplemented with *p*-coumaric acid (CM+C, black bars). Data were normalized with the *gyrA* gene which was used as an internal control. Data shown are the mean of three replicates. Data with the same letter are not significantly different (*P* < 0.05, LSD test).

The expression of seven genes associated with the degradation of aromatic compounds functional group (Table 3) and of three additional genes that were identified using basic local alignment search tool (BLAST) from *S. scabies* 87.22 genome was investigated. The three additional genes were SCAB_15331 (a putative IclR-family transcriptional regulator gene located in the vicinity of SCAB_15301 and SCAB_15321), SCAB_2141 (2,3-dihydroxy-2,3-dihydro-phenylpropionate dehydrogenase encoding gene) and SCAB_13661 (3-oxoadipate enol-lactone hydrolase-encoding gene). All genes tested were overexpressed in the presence of at least one of the two cinnamic acids (*trans*-ferulic acid or *p*-coumaric acid) (Figure 4).

SCAB_15601 and SCAB_15591 code for a putative feruloyl-CoA synthetase and a putative enoyl-CoA hydratase, respectively. They are potentially responsible for the transformation of ferulic acid into vanillic acid. Their gene expression was increased over 100- and 300-fold in the presence of ferulic and coumaric acids, respectively (Figure 4). The expression of the three genes identified as potential candidates for the conversion of vanillate into protocatechuate showed between 15-fold (SCAB_15331) and 300-fold overexpression (SCAB_15301 and SCAB_15321) in the presence of *trans*-ferulic acid (Figure 4). In contrast, the relative expression of these three genes was not statistically different when the bacteria were grown in the control medium supplemented or not with *p*-coumaric acid.

SCAB_2141 expression was found to be induced by *p*-coumaric acid but not by *trans*-ferulic acid. SCAB_2141 showed high expression in the presence of *p*-coumarate (ca. 20-fold upregulated). Whereas, no difference was recorded when *trans*-ferulic acid was added to the control medium (Figure 4).

**FIGURE 5:**
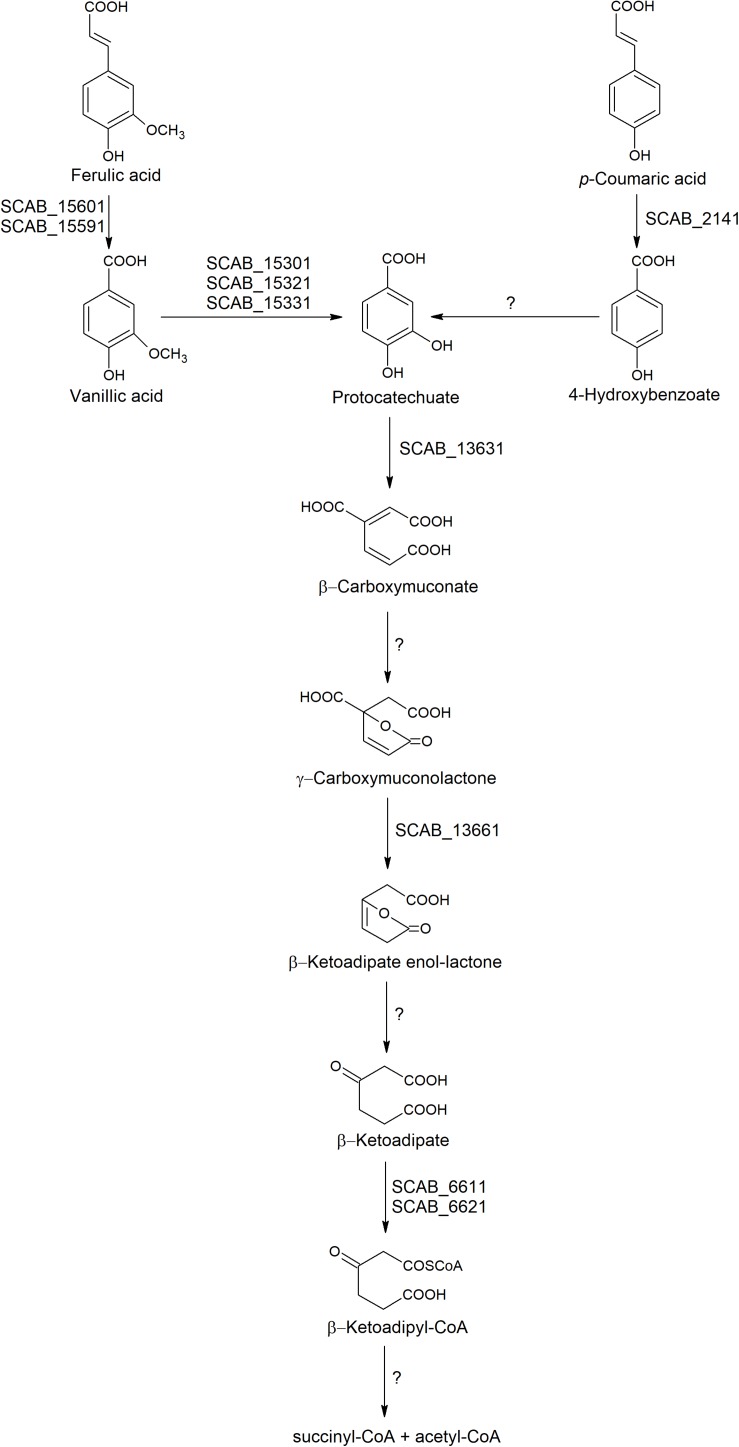
Hypothetic degradation pathway of *trans*-ferulic and *p*-coumaric acids in *S. scabies* 87.22.

The four genes involved in the main β-ketoadipate pathway that were tested showed very similar expression patterns toward both substrates. SCAB_13631 showed a relative expression increasing from 40- to 60-fold in the presence of *p*-coumaric and *trans*-ferulic acid, respectively. SCAB_13661, a gene predicted to be responsible for conversion of γ-carboxymuconolactone into β-ketoadipate enol-lactone, showed a 36-fold overexpression when the control medium was supplemented with *trans*-ferulic acid and a 27-fold overexpression with *p*-coumarate. SCAB_6611 showed higher expression in the presence of *trans*-ferulic acid when compared to the presence of *p*-coumaric acid. SCAB_6621, which is predicted to further metabolize β-ketoadipate into β-ketoadipyl-CoA, showed around 30-fold overexpression with both substrates when compared to the control medium. A hypothetical degradation pathway of *trans*-ferulic and *p*-coumaric acids is proposed (Figure 5).

To determine if *trans*-ferulic or *p*-coumaric acid or one of their degradation products were the inducers of the β-ketoadipate pathway, the expression of SCAB_13631 (the first gene in the proposed β-ketoadipate pathway) was tested in presence of *trans*-ferulic, *p*-coumaric or protocatechuic acids. The gene SCAB_13631 showed around 60-fold overexpression in the presence of *trans*-ferulic acid in the wild strain. Addition of *trans-*ferulic acid in the culture medium of the deletion mutant strain ΔSCAB_15301 did not significantly induce transcription of gene SCAB_13631 (relative expression of 1.1 ± 0.31). SCAB_13631 showed similar expression rate in the presence of coumarate (42.36 ± 4.18 and 44.3 ± 3.9, respectively) or protocatechuate (4.05 ± 0.38 and 4.23 ± 0.97, respectively) in the wild strain and the deletion mutant.

### Effects of Suberin on the Expression of the Putative Aromatic Compounds Degradation Genes

**FIGURE 6:**
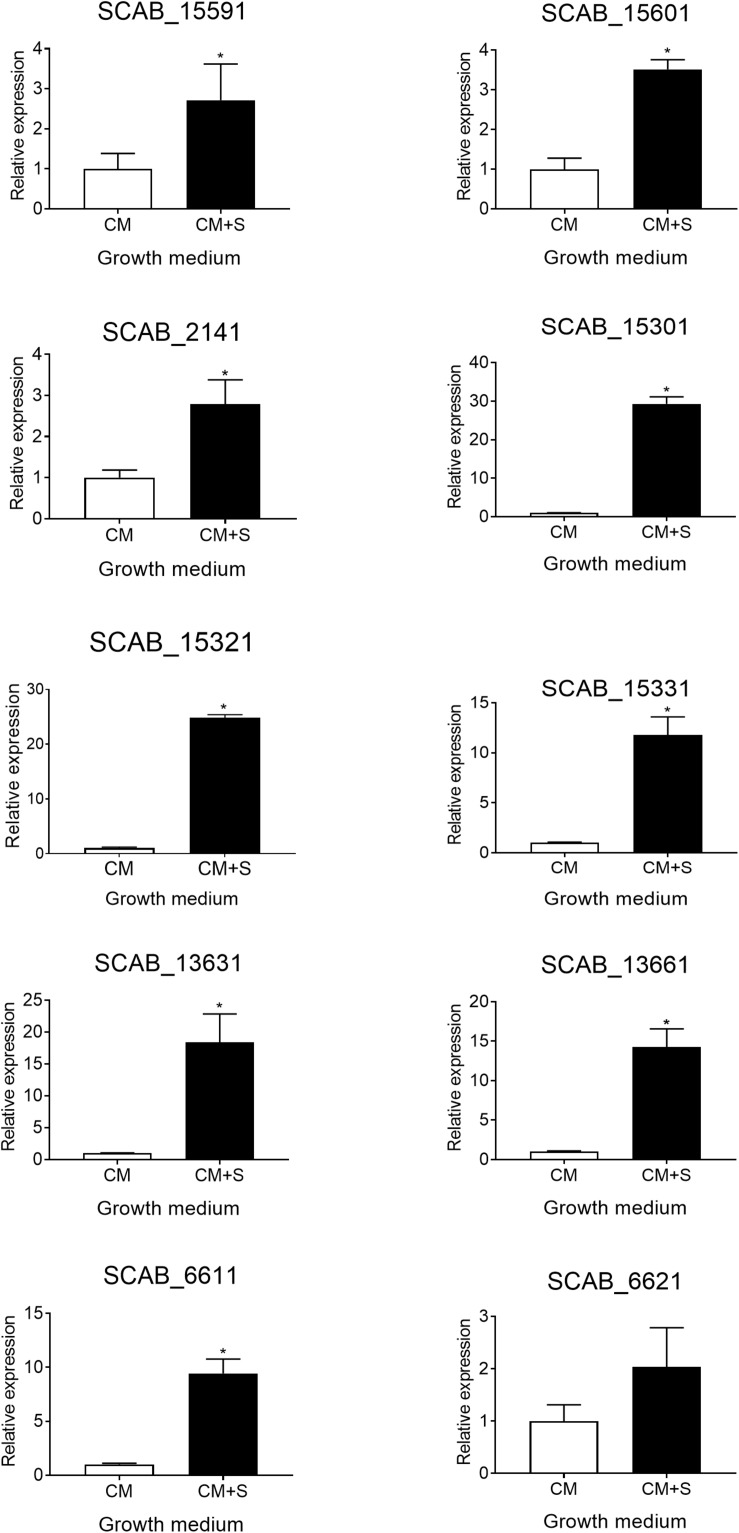
Relative expression levels (+SD) of targeted genes involved in *trans*-ferulic and *p*-coumaric acids degradation from *S. scabies* 87.22 grown in control medium (CM) alone and control medium (CM) supplemented with suberin (CM+S, black bars). Data were normalized with the *gyrA* gene which was used as an internal control. Data shown are the mean of three replicates. Data with ^∗^ are significantly different from the control (*P* < 0.05, *t*-test).

The expression of the genes predicted to be involved in the degradation of aromatic compounds (see above) was tested in the presence of suberin-enriched potato periderm. All tested genes, except gene SCAB_6621, showed a higher level of expression when *S. scabies* 87.22 was grown in the presence of suberin (between 2- and 30-fold overexpression) (Figure 6).

### Vanillate Accumulation in the Mutant Culture Media

**FIGURE 7:**
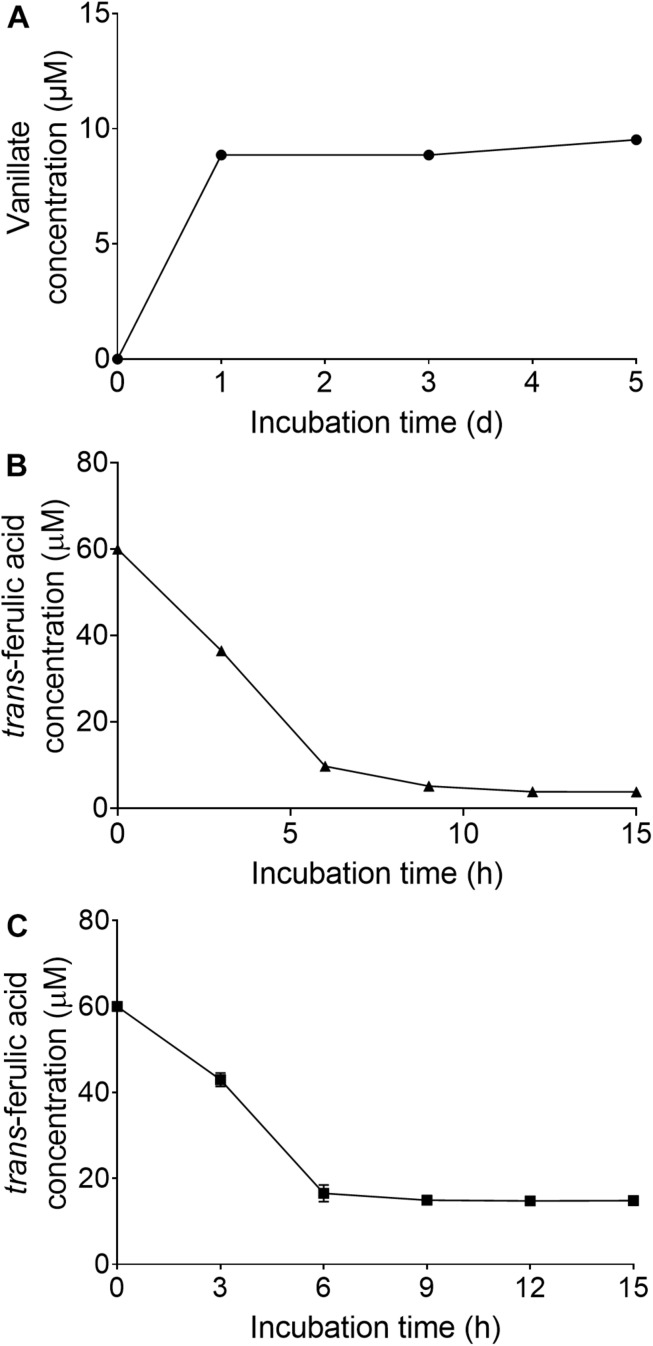
**(A)** Vanillate accumulation from suberin-enriched potato periderm in ΔSCAB_15301 (no accumulation was detected with the wild-type or the complemented strain). **(B)** Utilization of *trans*-ferulic acid in the ΔSCAB_15301 when grown in the presence of *trans*-ferulic acid. **(C)** Utilization of *trans*-ferulic acid in the ΔSCAB_15301 when grown in the presence of both *trans*-ferulic acid and vanillate. Data shown are the mean of three replicates (±SD).

When the wild strain *S. scabies* 87.22, deletion mutant ΔSCAB_15301 and complemented strain *S. scabies* com15301 were grown in the presence of suberin-enriched potato periderm, production of vanillic acid was observed only in the mutant culture medium (Figure 7A). The accumulation of vanillic acid from the suberin-enriched potato periderm rapidly reached a plateau (Figure 7A). Accumulation of vanillate may interfere with ferulic acid degradation as suggested by the fact that strain ΔSCAB_15301 was less efficiently catabolizing ferulic acid when grown in the presence of both *trans-*ferulic and vanillic acids (Figure 7C) than with ferulic acid alone (Figure 7B).

## Discussion

Beaulieu et al. (2016) previously demonstrated the ability of *S. scabies* to degrade the aliphatic moiety of suberin, but there has been no report about the degradation of the aromatic part of suberin by this pathogen. Nevertheless, a previous proteomic analysis revealed the presence of proteins predicted to play a role in the degradation of aromatic compounds when *S. scabies* was grown in the presence of suberin (Komeil et al., 2014). This study aimed to demonstrate the ability of *S. scabies* strains to utilize *trans*-ferulic and *p*-coumaric acids which are the main constituents of the suberin aromatic moiety.

In this study, *S. scabies* strains EF-35 and 87.22 were grown in a control medium supplemented with *trans*-ferulic and *p*-coumaric acids. Results revealed the ability of the two strains to efficiently degrade both substrates. The ability to degrade these cinnamic acids is shared by other streptomycetes such as *S. setonii* (Sutherland et al., 1983), *Streptomyces* sp. strain V-1 (Yang et al., 2013), *Streptomyces sannanensis* (Ghosh et al., 2007), *Streptomyces canus* GLY-P2 (Wu et al., 2019), *Streptomyces coelicolor* and *Streptomyces viridosporus* (Davis and Sello, 2010).

Sutherland et al. (1983) reported the ability of *S. setonii* to catabolize ferulic acid into vanillin, vanillic acid, and protocatechuic acid. The proteomic study of *S. scabies* 87.22 in the presence of ferulic acid allowed the identification of proteins that could also be involved in the conversion of ferulic acid into vanillate (a putative feruloyl-CoA hydratase and feruloyl-CoA synthetase) and further degradation of vanillate into protocatechuate (a putative vanillate monooxygenase, iron-sulfur oxidoreductase beta subunit and IclR-family transcriptional regulator). In contrast, *S. sannanensis* was shown to bioconvert ferulic acid into vanillate without further degradation into protocatechuic acid, possibly because of the absence of vanillic acid demethylase and vanillic acid decarboxylase activities in this bacterium (Ghosh et al., 2007).

SCAB_15591 and SCAB_15601 genes, encoding putative feruloyl-CoA hydratase and feruloyl-CoA synthetase, respectively, which convert *trans*-ferulic acid into vanillate, were highly expressed in the presence of both ferulic and coumaric acids. This could be attributed to their broad substrate specificity. Masai et al. (2002) identified those two genes in *Sphingomonas paucimobilis* SYK-6 and showed that these enzymes exhibited broad substrate specificity as they were able to degrade *p*-coumaric acid, caffeic acid, and sinapinic acid. On the other hand, three genes were identified in *S. scabies* to be potentially involved in the catabolism of vanillate (SCAB_15301, SCAB_15321, and SCAB_15331). The fact that those three genes were induced by ferulate suggests that they are involved in the conversion of vanillate into protocatechuic acid. These genes were found to be induced in the presence of ferulic acid but not coumaric acid, suggesting their substrate specificity.

The predicted function of SCAB_15301 (vanillate monooxygenase) was confirmed by the fact that the mutant degraded *trans*-ferulic and accumulated vanillate as a degradation product. By contrast, in the wild-type strain, no accumulation of vanillic acid was detected when grown in the presence of *trans*-ferulic acid. Absence of ferulic acid degradation product suggests further metabolism of ferulate in *S. scabies*.

Previous work showed that when *p*-coumaric acid was used as an initial growth substrate for *S. setonii*, it was catabolized into *p*-hydroxybenzaldehyde, *p*-hydroxybenzoic acid and protocatechuic acid (Sutherland et al., 1983). SCAB_2141 gene showed sequence homology with 2,3-dihydroxy-2,3-dihydro-phenylpropionate dehydrogenase, suggesting that this gene could be responsible for the conversion of *p*-coumaric acid into *p*-hydroxybenzoic acid. The fact that this gene was induced by coumaric acid but not by ferulic acid also supports this metabolic function.

Some proteins found in the proteome of *S. scabies* 87.22 cultured in the presence of ferulic acid were predicted to be involved in the catabolism of protocatechuate through the β-ketoadipate pathway (protocatechuate 3,4-dioxygenase beta subunit, 3-oxoadipate CoA-transferase subunit and 3-oxoadipate CoA-transferase subunit A). The β-ketoadipate pathway is a chromosomally encoded pathway that is widely distributed in soil bacteria and fungi (Harwood and Parales, 1996). This highly conserved pathway has two branches; one of them converts protocatechuate, derived from phenolic compounds such as ferulic and coumaric acids, to β- ketoadipate. The gene cluster for protocatechuic acid catabolism was characterized in *Streptomyces* sp. strain 2065 (Iwagami et al., 2000). In this strain, a protocatechuate 3,4-dioxygenase was purified and seven genes were identified to be involved in the degradation of protocatechuate via the β-ketoadipate pathway (Iwagami et al., 2000).

In several studies, it was reported that the expression of β-ketoadipate pathway genes was induced by aromatic compounds or by β-ketoadipate (Harwood and Parales, 1996). The high expression of genes involved in the β-ketoadipate pathway in the presence of both *trans*-ferulic and *p*-coumaric acids in *S. scabies* supports the hypothesis that these two phenolic compounds share a common degradation pathway (β-ketoadipate pathway). In ΔSCAB_15301, the gene SCAB_13631, which encodes the first key enzyme in the β-ketoadipate pathway, was not induced in the presence of ferulic acid, suggesting that neither ferulic nor vanillic acid was the real inducer of the β-ketoadipate pathway. However, SCAB_13631 was induced in the presence of protocatechuate in both the mutant and the wild-type strains, suggesting that protocatechuate or its degradation products are the real inducers of the pathway. These results are in accordance with Davis and Sello (2010) who showed that both *S. coelicolor* and *S. viridosporus* contained the *pca* structural genes. These *pca* genes encode the enzymes of the protocatechuate branch of the β-ketoadipate pathway and were shown to be induced by protocatechuate and by *p*-hydroxybenzoate.

Most of the genes identified as being involved in degradation of aromatic compounds showed high expression in the presence of potato suberin, which could be attributed to the hydroxycinnamates content of suberin (Bernards et al., 1995; Bernards and Lewis, 1998). However, their expression in the presence of suberin was much lower than in the presence of ferulic and coumaric acids. This could be due to the fact that suberin degradation is a slow process (Hamer et al., 2012; Beaulieu et al., 2016). Vanillic acid accumulated when ΔSCAB_15301 was grown in the presence of suberin, suggesting that *S. scabies* 87.22 can retrieve ferulic acid from potato periderm. Similarly, previous work revealed the ability of another actinobacterium, *S. cinnamoneus*, to release ferulic acid from biomass (Uraji et al., 2018).

Phenolic compounds were shown to induce stress in bacteria. *p*-coumaric acid can disturb protein structure, affect the properties of the cell membrane and interfere with DNA replication of *Lactobacillus casei* BL23 (Rivas-Sendra et al., 2011). The proteome analysis of *S. scabies* 87.22 in the presence of ferulic acid revealed the production of stress proteins, especially protein involved in acid stress tolerance. Among these proteins is a uracil-DNA glycosylase, which has been reported to be involved in DNA repair, mutation prevention and tolerance to acidified nitrite in G+C-rich bacteria (Venkatesh et al., 2003). An aspartate ammonia-lyase (AspA) was also detected. This protein was shown to increase the acid survival in *Yersinia pseudotuberculosis* by producing ammonia (Hu et al., 2010). The α-acetolactate synthase (ALS) may contribute to *S. scabies* pH homeostasis following the addition of ferulic acid as an α-acetolactate synthase played this function in *Lactococcus lactis* under acid stress conditions (Zuljan et al., 2014). The most abundant protein found in the presence of ferulic acid only is a putative betaine aldehyde dehydrogenase. Since betaine is known to be an osmolyte produced in response to stress, especially to osmotic stress (Park et al., 1995), the induction of betaine aldehyde dehydrogenase in the presence of ferulic acid may represent a response to general stress. However, streptomycetes have been shown to produce several NADPH/NADH-producing enzymes in presence of recalcitrant compounds that could help to offset the low level of energy obtained from their catabolism (Sineli et al., 2018). These findings reveal that *S. scabies* has undergone various adaptive physiological mechanisms in response to ferulic acid.

*Streptomyces scabies* also produced proteins involved in lipid metabolism after the addition of ferulic acid to its growth medium. In potato periderm, ferulate esters are important structural elements but represent only a small fraction of suberin constituents which is mainly composed of fatty acids (Beaulieu et al., 2016). Therefore, ferulic acid might act as a signal to produce enzymes such as those of the fatty acids oxidation complex. This protein complex which breaks down CoA activated derivatives of fatty acids into acetyl-CoA by the β-oxidation pathway (Menendez-Bravo et al., 2017) might be important for the degradation of the aliphatic constituents of suberin. Interestingly, the second most abundant protein induced by the addition of ferulic acid, SCAB_3954, is encoded by a gene of unknown function that is located in a gene cluster dedicated to Coenzyme A biosynthesis. Such relationship between fatty acid metabolism and catabolism of aromatic compounds has also been suggested in another actinobacterium, *Rhodococcus* sp. strain BUBNP1. A transcriptomics study revealed that strain BUBNP1 degrades concurrently 4-nitrophenol and fatty acids (Sengupta et al., 2019).

Our study indicates that *S. scabies* can utilize hydroxycinnamates, contained in suberin, as carbon and energy sources via the β-ketoadipate pathway. This may be advantageous in environments where carbon sources are limited. In the last step of the β-ketoadipate pathway, once β-ketoadipyl-CoA is formed, it is converted to succinyl-CoA and acetyl-CoA. The latter two compounds are further metabolized via Krebs cycle, yielding energy. This could offer not only an ecological advantage to *S. scabies* as a saprophyte but also as a pathogen. Further research is required to elucidate the importance of degradation of aromatic compounds via β-ketoadipate in *S. scabies* environment, as it was reported before that the catabolism of aromatic compounds through β-ketoadipate pathway is necessary for the pathogenicity of other plant pathogens such as *Fusarium oxysporum* (Michielse et al., 2012).

## Data Availability Statement

All datasets generated for this study are included in the article/Supplementary Material.

## Author Contributions

CB and NB conceived and designed the experiments. MK and SL performed the lab work and analyzed the data. MK and CB wrote the manuscript with the critical review of NB and SL. CB supervised the project. All authors approved the final version of the manuscript.

## Conflict of Interest

The authors declare that the research was conducted in the absence of any commercial or financial relationships that could be construed as a potential conflict of interest.
