# Supplementary material for: The Plant Pathogenic Bacterium Streptomyces scabies Degrades the Aromatic Components of Potato Periderm via the β-Ketoadipate Pathway
Source: Front Microbiol. 2019 Dec 4;10:2795. doi: 10.3389/fmicb.2019.02795 (PMC6904314; doi:10.3389/fmicb.2019.02795)
Supplement: Supplementary file 1 [file Data_Sheet_1.PDF]

Supplementary material for:

The plant pathogenic bacterium *Streptomyces scabies* degrades the aromatic components of potato periderm via the β-ketoadipate pathway

Mario Khalil[1,2], Sylvain Lerat[1], Nathalie Beaudoin[1], Carole Beaulieu[1*]

[1]Centre SÈVE, Département de Biologie, Université de Sherbrooke, Sherbrooke, QC, Canada

[2]Department of Microbiology, Faculty of Science, Ain Shams University, Cairo, Egypt

*** Correspondence:**
Carole Beaulieu
carole.beaulieu@usherbrooke.ca

```
C9ZBH8(SCAB_15301)  ------------------------------------MTAFARNQWYVAAYAEEVGR-ELLGRTILGEPLVFYRTEEEGTPVALHDRCVHRRYPLSK--SG
O88036              ---------------------------------MPHMTAFARNQWYVAAYSHEVGR-ELLGRTVLGEPLVLYRAEEDGGPVVLHDRCVHRRYPLSEAPTR
A0A0A0M872          ---------------------------MTGVTTLARMTAFAKNQWYVAAYGREIGR-ELLGRTILDEPIAFYRTESDDKVIALADRCVHRRYPLTA--GR
A0A385ZE35          ---------------------------------MPHMTAFARNQWYVAAYSHEVGREELLGRTVLGEPLVFYRTEEDGTPVALADRCVHRRFPLHEKPSR
A0A0F7VMK6          MPPGGFREAEGVLRRRAALSDDVGTTRGREPEAMPHMTAFARNQWYVAAYAHEVGR-ELLGRTILGEPLVLYRTR-EGTPVVLHDRCVHRRYPLSEAPTR
C7Q1M6              -----------------------------------MATAFARNQWYVAAYASEVGR-TFLARTILGEPIVFYRTGQDGRAVALADRCVHRRYPLSE--SR
                                                         ****:********. *:**  :*.**:*.**:.:**:  :.  :.* *******:**

C9ZBH8(SCAB_15301)  LDGDRIVCGYHGFTYDTTGACVYVPGQKRIPRTARVASYPVVEQDSLIWVWIGDPALADPQTIPRARHLAAPGWTTVRGMEPIDADYGLLVDNLLDLSHE
O88036              LDGDRIVCGYHGFTYDTTGTCVYVPGQKRVPRTARVASYPVAERDSLVWVWIGDPALADPEAVPRARHLDAPGWVTVRGMEPIDADYGLLVDNLLDLSHE
A0A0A0M872          LDGDTVVCGYHGFTYDTGGTCVFVPGQKRVPRTARVRSYPVVEQDSLVWVWIGDPALADPGALPRAPWLADPRWTTVGGMEPIDADYGLLVDNLLDLSHE
A0A385ZE35          LDGDKLVCGYHGFTYDTTGTCVYVPGQKRVPRTARVASYPVVEQDSLVWVWIGDPALADADTIPRARHLDSPGWVTVRGMEPIDADYGLLVDNLLDLSHE
A0A0F7VMK6          LDGDRIVCGYHGFTYDTTGTCVAVPGQKRVPRTARVPSYPVVEQDSLVWVWIGDPARAEPRAIPRAPHLDSPGWVTVRGMEPIDCDYGLLVDNLLDLSHE
C7Q1M6              LDGDTIVCGYHGFTYDTSGTCVFVPGQQRIPRTARVASYPVAELDSFVWVWIGDPELADDKLIPRAPHMADPEFVTVSGMEPIDCDYGLLVDNLLDLSHE
                    **** :*********** *:** ****:*:****** ****.* **::*******  *:   :***  :  * :.** ******.***************

C9ZBH8(SCAB_15301)  TYLHGGYIGTPEVAETPITTEVDEGAGVVRVSRHMDDAECPPFYARSTGIEGRITRWQDIEYFAPCLYLLHSRIAPVGVLPEADGSDPNGFHTEITYAIT
O88036              TYLHGGHIGTPEVAETPITTEVDEGAGIVRVSRHMDDAECPPFYARSTGIVGRIDRWQDIEYHAPCLYVLHSRVAPTGTVPAPDGGDPEGFHTEITYAIT
A0A0A0M872          TYLHGGYIGTPEVAETPITTEVDEGAGVVRVSRHMDDAECPPFYARSTGIEGRITRWQDIEYHAPCLYLLHSRIAPVGVLPEPDGSDPNAFHVEITYAIT
A0A385ZE35          TYLHGGYIGTPEVAETPITTEVDEGAGIVRVSRHMDDAACPPFYAKSTGIEGRITRWQDIEYHAPCLYLLHSRIAPVGVLPEADGSDPNGFHTEITYAIT
A0A0F7VMK6          TYLHGGYIGTPEVAETPITTEVDEGAGTVRVSRHMDDAECPPFYAESTGIQGRISRWQDIEYHAPCLYLLHSRVAPVGVLPAADGSDPDGFHTEITYAIT
C7Q1M6              TYLHGGYIGTPEVADTPITTDADEQAGIVRVARHMVDAACPPFYAKSTGIQGRITRLQDIEYFAPCLYLLHSRITPAGE-------QNPLFRTEITYAIT
                    ******:*******:*****:.** ** ***:*** ** ******.**** *** * *****.*****:****::*.*        :   *:.*******

C9ZBH8(SCAB_15301)  PSSDGKVYDFWMVSRDWATESDEVTEFLRGNNHTVVMQDVVALNLLQETLGSERTGYQELSINIDTGGLAARRILARLVEQGEKPVEKVL---
O88036              PSGDGKVYDFWAVSRDWATDDAEVTEFLYKSNRTVVMQDVDALNLLQRTLGGERAGYQELSINIDTGGLAARRILARLVEEGEGAGTGPRVAR
A0A0A0M872          PSTDHHVYDFWAVSRDFAQDDEEVSTFLHDLNRTVVLQDVDALNVLQKALDTEREGYQELSINIDTGGLAARRILARLAAEGEKTAPVVAAK-
A0A385ZE35          PSADGHVYDFWMVSRDWATEDTEVTEFLRGNNHTVVMQDVDALNLLQRTLGTERAGYQELSINIDTGGLAARRILARLVEEGDKPVEKVL---
A0A0F7VMK6          PSADGRVYDFWAVSRDWATDDADVTEFLRKNNHTVVMQDVTALNLLQRTLGTERTGYQELSINIDTGGLAARRILARLAEEGAGAAEPVEGVR
C7Q1M6              PSAPGQVYDFWAVSRNFATDDPAVTEFLRDFNHQVVMQDVVALNILQKALDSESAGYQELSIGIDAGGLAARRILAQLAAQ------------
                    **   :***** ***::* :.  *: **   *: **:*** ***:**.:*. *  *******.**:**********:*. :
```

Supplementary Fig. S1. Alignment of the predicted protein of SCAB_15301 (UnibProt #C9ZBH8) with protein sequences of known functions from related actinobacterial species. O88036: *Streptomyces coelicolor* vanillate O-demethylase monooxygenase subunit, A0A0A0N872: *Streptomyces rapamycinicus* vanillate monooxygenase, A0A385ZE35: *Streptomyces griseorubiginosus* toluene-4-sulfonate monooxygenase system iron-sulfur subunit, A0A0F7VMK6: *Streptomyces leeuwenhoekii* vanillate O-demethylase oxygenase subunit, C7Q1M6: *Catenulispora acidiphila* vanillate monooxygenase. Sequence alignment was performed using the Clustal Omega software.

Supplementary Table 1. Proteins produced by *Streptomyces scabies* 87.22 during growth in CM supplemented or not with *trans*-ferulic acid.

Uniprot accession #	Corresponding gene in *S. scabies* 87.22	Putative protein function	NSpC-C	NSpC-F
**Carbohydrate transport and metabolism**				
C9YW88	SCAB_37051	Beta-xylanase	0.45	0.40
C9Z433	SCAB_42161	Fructose 1,6-bisphosphate aldolase	0.28	0.49
C9ZGR4	SCAB_34111	Phosphoenolpyruvate carboxykinase	0.16	0.22
C9Z6V6	SCAB_27951	Glycogen phosphorylase	0.07	0.04
C9Z9C4	SCAB_76231	Mannose-1-phosphate guanyltransferase	0.12	0.12
C9YY64	SCAB_69721	Triosephosphate isomerase	0.06	0.09
C9ZF65	SCAB_33521	Fructose-1,6-bisphosphatase	0.34	0.32
C9ZAM6	SCAB_46011	1L-myo-inositol-1-phosphate synthase	0.17	0.24
C9ZDV4	SCAB_63781	Alpha-L-arabinofuranosidase	0.05	0.06
C9YY84	SCAB_69921	Transketolase	0.11	0.15
C9YTW6	SCAB_67551	Glucokinase	0.14	0.14
C9YUT7	SCAB_35801	Succinate--CoA ligase	0.17	0.15
C9Z5N4	SCAB_43191	Hydrolase	0.33	0.37
C9YU13	SCAB_81861	Pyruvate carboxylase	0.02	0.07
C9YY62	SCAB_69701	Glyceraldehyde-3-phosphate dehydrogenase	0.40	0.33

C9YUT6	SCAB_35791	Succinyl-CoA ligase	0.15	0.20
C9YT49	SCAB_19421	Oxidoreductase	0.08	0.07
C9ZH18	SCAB_49941	2,3-bisphosphoglycerate-dependent phosphoglycerate mutase	0.23	0.20
C9Z545	SCAB_11811	6-phosphogluconate dehydrogenase, decarboxylating	0.14	0.13
C9YY63	SCAB_69711	Phosphoglycerate kinase	0.13	0.12
C9ZH59	SCAB_50381	Trehalose-phosphate synthase	0.12	0.15
C9Z4A0	SCAB_58271	Sugar hydrolase	0.17	0.15
C9YY82	SCAB_69901	Glucose-6-phosphate 1-dehydrogenase	0.17	0.13
C9ZBJ5	SCAB_15481	Alpha-mannosidase	0.08	0.09
C9Z109	SCAB_55931	Malate dehydrogenase	0.24	0.21
C9Z737	SCAB_43661	Secreted protein	0.20	0.19
C9Z5R8	SCAB_58791	Citrate synthase	0.36	0.30
C9YY67	SCAB_69751	Glucose-6-phosphate isomerase (GPI)	0.14	0.10
C9ZAT6	SCAB_62141	Pyruvate phosphate dikinase	0.22	0.26
C9YY83	SCAB_69911	Transaldolase	0.49	0.48
C9ZFY5	SCAB_79861	Xylose isomerase	0.43	0.36
C9YY37	SCAB_54441	Enolase	0.69	0.68
**Stress mechanism**				
C9Z7C3	SCAB_59681	Secreted peptidase	0.08	0.09

C9ZE08	SCAB_64341	Stress protein	1.03	0.78
C9Z7C8	SCAB_59731	Superoxide dismutase	0.48	0.48
C9ZEQ9	SCAB_17101	ATP/GTP binding protein	0.25	0.18
C9ZAA4	SCAB_29931	Nickel superoxide dismutase	0.57	0.57
C9Z721	SCAB_43491	Uncharacterized protein	0.40	0.33
C9YYC2	SCAB_70311	Stress-induced protein	0.58	0.61
C9Z785	SCAB_44161	Hydrolase	0.33	0.23
C9ZAL8	SCAB_45931	Thioredoxin reductase	0.40	0.35
C9Z1W0	SCAB_9531	Catalase-peroxidase	0.37	0.34
C9YWU0	SCAB_69441	Stress-induced protein	0.79	0.52
C9ZE07	SCAB_64331	Stress protein	2.33	1.58
C9ZH47	SCAB_50261	Stress protein	3.00	2.00
C9ZHS9	SCAB_81661	Tellurium resistance protein	2.58	1.98
**Lipid metabolism**				
C9YTK3	SCAB_51091	Secreted peptidase	0.61	0.44
C9Z707	SCAB_28481	Acetyl-coA C-acetyltransferase FadA	0.52	0.54
C9ZAG3	SCAB_45341	3-hydroxyacyl-CoA dehydrogenase	0.16	0.13
C9ZGV4	SCAB_34521	Enoyl-CoA hydratase	0.36	0.17
C9Z5Z2	SCAB_74351	Secreted glycerophosphoryl diester phosphodiesterase	0.20	0.17

C9YZS0	SCAB_70981	Enoyl-CoA hydratase/isomerase	0.09	0.09
C9Z6U1	SCAB_27801	Enoyl-coA hydratase/isomerase family protein	0.24	0.13
C9ZDY7	SCAB_64131	3-oxoacyl-[acyl-carrier-protein] synthase 2	0.07	0.07
C9Z0I2	SCAB_24201	Uncharacterized protein	0.31	0.38
C9Z9F5	SCAB_76541	3-oxoacyl-[acyl-carrier protein] reductase	0.15	0.19
C9Z8D6	SCAB_28641	3-hydroxybutyryl-coA dehydrogenase	0.23	0.22
C9Z1F6	SCAB_72321	Secreted lipase	0.10	0.28
C9Z1E2	SCAB_72171	Acyl-CoA dehydrogenase	0.12	0.09
C9YYH5	SCAB_83811	Dihydroxyacetone kinase component	0.10	0.09
C9YZG6	SCAB_54931	Acyl-CoA dehydrogenase	0.10	0.12
C9Z4I9	SCAB_74071	3-hydroxyacyl-CoA dehydrogenase	0.15	0.20
C9ZDZ0	SCAB_64161	Malonyl CoA:acyl carrier protein malonyltransferase	0.39	0.31
C9YX75	SCAB_6631	Acyl-CoA thiolase	0.06	0.20
C9Z2I3	SCAB_41241	Acetyl-coenzyme A synthetase	0.13	0.09
C9YZV5	SCAB_71331	Enoyl-[acyl-carrier-protein] reductase [NADH]	0.31	0.28
C9Z6Y6	SCAB_28271	Cholesterol esterase	0.70	0.63
C9YY49	SCAB_54571	Acetyl-CoA C-acyltransferase	0.34	0.44
**Transcriptional, translational, ribosomal structure and biogenesis**				
C9YWA2	SCAB_37191	DNA-directed RNA polymerase subunit beta	0.37	0.37

C9Z240	SCAB_25251	Polyribonucleotide nucleotidyltransferase	0.59	0.53
C9YW51	SCAB_36671	DNA-directed RNA polymerase subunit alpha	0.97	1.07
C9YW66	SCAB_36821	50S ribosomal protein L5	1.38	1.50
C9YW75	SCAB_36911	50S ribosomal protein L2	0.83	0.78
C9YW64	SCAB_36801	30S ribosomal protein S8	1.96	1.96
C9YW72	SCAB_36881	30S ribosomal protein S3	0.82	0.78
C9YW78	SCAB_36941	50S ribosomal protein L3	0.76	0.80
C9ZE09	SCAB_64351	Integral membrane protein	0.21	0.20
C9Z7K6	SCAB_75261	Uncharacterized protein	1.38	1.00
C9Z241	SCAB_25261	30S ribosomal protein S15	1.32	1.23
C9Z3N7	SCAB_25991	Ribosome-recycling factor	0.57	0.38
C9Z0H0	SCAB_24081	Two-component response regulator	0.38	0.38
C9YVQ3	SCAB_6061	Two-component system response regulator	0.38	0.28
C9Z4I0	SCAB_73981	50S ribosomal protein L20	0.71	0.71
C9ZAK6	SCAB_45811	Beta sliding clamp	0.26	0.24
C9ZAN3	SCAB_46101	30S ribosomal protein S6	1.00	0.82
C9YW59	SCAB_36751	50S ribosomal protein L15	0.63	0.59
C9YW70	SCAB_36861	50S ribosomal protein L29	1.38	1.06
C9YW74	SCAB_36901	30S ribosomal protein S19	0.86	0.91

C9YW53	SCAB_36691	30S ribosomal protein S13	0.61	0.71
C9Z629	SCAB_74751	Threonine--tRNA ligase	0.09	0.11
C9Z316	SCAB_73401	Uncharacterized protein	0.29	0.30
C9YW77	SCAB_36931	50S ribosomal protein L4	0.48	0.37
C9YW67	SCAB_36831	50S ribosomal protein L24	1.00	0.96
C9YW79	SCAB_36951	30S ribosomal protein S10	0.83	1.13
C9YW62	SCAB_36781	50S ribosomal protein L18	0.54	0.21
C9YW47	SCAB_36631	50S ribosomal protein L13	0.84	1.13
C9YW76	SCAB_36921	50S ribosomal protein L23	0.83	0.53
C9YW95	SCAB_37121	30S ribosomal protein S12	0.75	0.71
C9YZB2	SCAB_38941	Lysine--tRNA ligase	0.15	0.10
C9Z2I8	SCAB_56731	Ribonuclease PH (RNase PH)	0.25	0.37
C9Z3Q3	SCAB_26151	50S ribosomal protein L19	0.92	0.88
C9YW50	SCAB_36661	50S ribosomal protein L17	0.94	0.81
C9YW93	SCAB_37101	Elongation factor G	0.20	0.26
C9YZB3	SCAB_38961	Arginine--tRNA ligase	0.10	0.14
C9Z4S3	SCAB_87641	Ribonuclease J	0.13	0.15
C9ZAN4	SCAB_46111	Single-stranded DNA-binding protein	0.55	0.34
C9YWV2	SCAB_69561	UvrABC system protein A	0.04	0.04

C9Z3N9	SCAB_26011	Elongation factor Ts	0.35	0.45
C9YW63	SCAB_36791	50S ribosomal protein L6	0.97	1.13
C9Z2D8	SCAB_40781	DNA topoisomerase 1	0.17	0.22
C9YW52	SCAB_36681	30S ribosomal protein S11	0.57	0.57
C9YZ53	SCAB_38351	AsnC-family transcriptional regulator	0.38	0.28
C9YW61	SCAB_36771	30S ribosomal protein S5	0.95	1.00
C9YWA6	SCAB_37231	50S ribosomal protein L1	0.15	0.27
C9YWA4	SCAB_37211	50S ribosomal protein L10	0.29	0.42
C9Z8T7	SCAB_44991	Aspartate--tRNA ligase	0.09	0.11
C9ZAK2	SCAB_45761	DNA gyrase subunit B	0.07	0.10
C9YW73	SCAB_36891	50S ribosomal protein L22	0.73	0.38
C9YWP5	SCAB_68981	DNA polymerase I	0.05	0.06
C9Z2E6	SCAB_40861	Anti-sigma factor antagonist	0.42	0.29
C9Z7H2	SCAB_60151	50S ribosomal protein L21	0.58	0.46
C9Z7J7	SCAB_75171	BldD regulator of morphogenesis and anitbiotic production	0.28	0.33
C9Z7Q6	SCAB_75801	AsnC-family transcriptional regulatory protein	0.22	0.25
C9YV68	SCAB_52291	Chitinase-promoter-binding protein	0.17	0.12
C9Z0W7	SCAB_40011	50S ribosomal protein L28	0.50	0.38
C9YWA3	SCAB_37201	50S ribosomal protein L7/L12	0.23	0.31

C9Z257	SCAB_25421	Proline--tRNA ligase	0.06	0.06
C9ZAN5	SCAB_46121	30S ribosomal protein S18	0.39	0.44
C9YW68	SCAB_36841	50S ribosomal protein L14	1.58	1.65
C9YZR9	SCAB_70971	Uncharacterized protein	0.75	0.56
C9YV23	SCAB_51821	DNA-binding protein	0.17	0.11
C9ZAN6	SCAB_46131	50S ribosomal protein L9	0.19	0.31
C9YY11	SCAB_54151	50S ribosomal protein L25	0.21	0.21
C9YW71	SCAB_36871	50S ribosomal protein L16	0.47	0.38
C9Z8G5	SCAB_28931	Transcription termination factor Rho	0.04	0.05
C9Z978	SCAB_61451	30s ribosomal protein S20	0.44	0.33
C9Z3V0	SCAB_41311	Cyclic-nucleotide-binding protein	0.30	0.20
C9YW46	SCAB_36621	30S ribosomal protein S9	1.34	1.11
C9YW69	SCAB_36851	30S ribosomal protein S17	2.09	2.14
C9Z7H3	SCAB_60161	50S ribosomal protein L27	0.39	0.33
C9ZAK1	SCAB_45751	DNA gyrase subunit A	0.26	0.29
C9Z459	SCAB_57831	A-factor-responsive transcriptional activator	0.08	0.08
C9YWA8	SCAB_37251	Transcription termination/antitermination protein NusG	0.20	0.16
C9ZAC1	SCAB_30101	Sigma factor	0.06	0.06
C9Z2B7	SCAB_40551	Cysteine--tRNA ligase	0.15	0.11

C9Z233	SCAB_25181	Lactamase-b family hydrolase	0.48	0.43
C9Z3V4	SCAB_41351	Endoribonuclease	0.31	0.34
C9YZC9	SCAB_39121	AdsA-like sigma factor	0.23	0.13
C9Z656	SCAB_75031	30S ribosomal protein S4	1.19	1.23
C9YWQ2	SCAB_69061	30S ribosomal protein S1	0.55	0.50
C9Z0J8	SCAB_24391	DNA topoisomerase (ATP-hydrolyzing)	0.04	0.05
C9YW94	SCAB_37111	30S ribosomal protein S7	1.76	1.59
C9Z0M0	SCAB_24631	Vitamin B12-dependent ribonucleotide reductase	0.05	0.07
C9Z3P0	SCAB_26021	30S ribosomal protein S2	1.24	1.22
C9YW92	SCAB_37091	Elongation factor Tu	0.68	0.97
C9YWA1	SCAB_37181	DNA-directed RNA polymerase subunit beta	0.57	0.54
**Nucleotide transport and metabolism**				
C9YWT5	SCAB_69391	Pseudouridine-5'-phosphate glycosidase	0.31	0.36
C9ZB78	SCAB_78051	Adenylosuccinate lyase	0.21	0.13
C9YWF6	SCAB_53071	Purine nucleoside phosphorylase	0.16	0.16
C9YZE5	SCAB_54711	Peptidase	0.07	0.08
C9YUT1	SCAB_35741	Bifunctional purine biosynthesis protein PurH	0.05	0.07
C9ZGX4	SCAB_49491	Secreted 5'-nucleotidase	0.17	0.13
C9ZDQ0	SCAB_47701	Phosphoribosylamine--glycine ligase	0.12	0.08

C9YZ58	SCAB_38401	3-octaprenyl-4-hydroxybenzoate carboxy-lyase	0.07	0.08
C9ZGW8	SCAB_34681	Purine nucleoside phosphorylase	0.26	0.24
C9Z7K4	SCAB_75241	Dihydroorotate dehydrogenase (quinone)	0.06	0.09
C9Z7F8	SCAB_60011	Nucleoside diphosphate kinase	1.00	0.83
C9Z5I3	SCAB_42681	dCTP deaminase	0.14	0.12
C9YVD1	SCAB_68051	Pyridoxal phosphate homeostasis protein	0.16	0.18
C9YVK8	SCAB_68841	5' nucleotidase	0.15	0.15
C9ZAW5	SCAB_62461	Deoxyguanosinetriphosphate triphosphohydrolase-like protein	0.09	0.09
C9YUX6	SCAB_36211	Inosine-5'-monophosphate dehydrogenase	0.36	0.45
C9Z407	SCAB_41891	Adenylosuccinate synthetase	0.30	0.29
C9Z995	SCAB_61621	Hit-family protein	0.38	0.38
C9YY13	SCAB_54171	Bifunctional protein GlmU	0.12	0.18
**Cell wall/membrane/envelope biogenesis**				
C9YV70	SCAB_52311	D-alanyl-D-alanine carboxypeptidase	0.07	0.07
C9Z111	SCAB_55951	UDP-N-acetylglucosamine 1-carboxyvinyltransferase	0.29	0.40
C9Z836	SCAB_13061	Nucleotide sugar-1-phosphate transferase	0.13	0.11
C9Z2W0	SCAB_72801	Secreted protein	0.14	0.17
C9Z8V2	SCAB_45141	D-alanyl-D-alanine carboxypeptidase	0.68	0.57
C9YT92	SCAB_34981	Lipoprotein	1.53	1.09

C9Z994	SCAB_61611	Protease	0.33	0.35
**Coenzyme transport and metabolism**				
C9Z3L8	SCAB_25801	Adenosine/AMP deaminase	0.15	0.11
C9Z7P0	SCAB_75631	6,7-dimethyl-8-ribityllumazine synthase	0.71	0.71
C9YYP1	SCAB_7581	Hydroxymethylbilane synthase	0.15	0.13
C9Z8E0	SCAB_28681	Cobalamin adenosyltransferase	0.17	0.14
C9YZQ8	SCAB_70851	Cob(I)yrinic acid a,c-diamide adenosyltransferase	0.14	0.14
C9YUS8	SCAB_35711	Bifunctional protein FolD	0.27	0.17
C9Z3T0	SCAB_26421	Phosphopantetheine adenylyltransferase	0.19	0.22
C9Z638	SCAB_74841	Pyridoxal 5'-phosphate synthase subunit PdxS	0.93	0.74
C9Z1Y4	SCAB_9771	Probable cobalamin biosynthesis protein cobN	0.51	0.47
C9ZBZ9	SCAB_46341	Phosphomethylpyrimidine synthase	0.07	0.07
C9Z3M4	SCAB_25861	Aminotransferase	0.29	0.29
**Amino acid transport and metabolism**				
C9YTK4	SCAB_51101	Phosphoserine aminotransferase	0.56	0.55
C9ZHG5	SCAB_66881	Glutamine synthetase	0.49	0.56
C9Z204	SCAB_24891	Glutamate uptake system binding subunit	1.50	1.34
C9Z7L0	SCAB_75311	S-adenosylmethionine synthase	0.36	0.35
C9ZAW6	SCAB_62471	Secreted aminopeptidase	0.30	0.25

C9Z7C5	SCAB_59701	Aminopeptidase	0.13	0.19
C9Z234	SCAB_25191	4-hydroxy-tetrahydrodipicolinate synthase	0.56	0.48
C9ZGG7	SCAB_18081	Gamma-glutamyltranspeptidase	0.13	0.12
C9Z7H9	SCAB_60221	Gamma-glutamyl phosphate reductase	0.26	0.27
C9ZGT9	SCAB_34371	Cystathionine gamma-synthase	0.30	0.27
C9Z9D4	SCAB_76331	Glycine dehydrogenase	0.20	0.23
C9Z5A0	SCAB_27061	Ketol-acid reductoisomerase	0.47	0.53
C9Z1B0	SCAB_71831	Alanine dehydrogenase	0.20	0.26
C9Z8G7	SCAB_28951	Threonine synthase	0.16	0.34
C9Z238	SCAB_25231	4-hydroxy-tetrahydrodipicolinate reductase	0.42	0.27
C9ZC37	SCAB_46731	Xaa-Pro aminopeptidase	0.45	0.35
C9Z443	SCAB_42271	Aspartate aminotransferase	0.18	0.16
C9Z483	SCAB_58101	Guanidinobutyrase	0.29	0.20
C9YTR8	SCAB_67071	Probable cytosol aminopeptidase	0.63	0.52
C9Z593	SCAB_26991	Aldehyde dehydrogenase	0.07	0.10
C9Z2Y0	SCAB_73011	Peptidase	0.06	0.08
C9Z4K3	SCAB_74211	Arginine biosynthesis bifunctional protein ArgJ	0.08	0.10
C9Z281	SCAB_25691	4-aminobutyrate aminotransferase	0.63	0.53
C9ZCM3	SCAB_78471	Ornithine aminotransferase	0.16	0.14

C9YY98	SCAB_70071	Cysteine desulfurase	0.17	0.14
C9ZC04	SCAB_46391	Phenylalanine aminotransferase	0.21	0.21
C9YYA5	SCAB_70141	2,3,4,5-tetrahydropyridine-2,6-dicarboxylate N-succinyltransferase	0.13	0.18
C9Z2X8	SCAB_72981	Secreted metallopeptidase	0.08	0.14
C9ZGG2	SCAB_18021	Uncharacterized protein	0.08	0.04
C9YZJ1	SCAB_55181	Adenosylhomocysteinase	0.16	0.21
C9Z6N6	SCAB_12671	2-amino-3-ketobutyrate coenzyme A ligase	0.12	0.10
C9Z4K2	SCAB_74201	N-acetyl-gamma-glutamyl-phosphate reductase	0.17	0.16
C9YYX2	SCAB_23141	Ornithine carbamoyltransferase	0.07	0.08
C9YVG2	SCAB_68371	Imidazoleglycerol-phosphate dehydratase	0.11	0.14
C9YYX3	SCAB_23151	Arginine deiminase	0.11	0.10
C9ZBY2	SCAB_31381	Dipeptidyl-peptidase IV	0.07	0.05
C9Z4G8	SCAB_73851	Glutamine synthetase	0.07	0.06
C9Z7B6	SCAB_59611	Aminopeptidase N	0.10	0.11
C9YVH4	SCAB_68491	Indole-3-glycerol phosphate synthase	0.16	0.11
C9Z8U5	SCAB_45071	Probable M18 family aminopeptidase 2	0.21	0.22
C9Z7P2	SCAB_75651	ATP phosphoribosyltransferase	0.19	0.19
C9ZAQ8	SCAB_61851	2-isopropylmalate synthase	0.04	0.06
C9ZHC2	SCAB_66451	Glutamine-dependent NAD(+) synthetase	0.19	0.17

C9ZGV2	SCAB_34501	Histidine ammonia-lyase	0.46	0.41
C9YWB0	SCAB_37271	Aspartate aminotransferase	0.32	0.31
C9YZE4	SCAB_54701	Urocanate hydratase	0.75	0.55
C9YWP0	SCAB_68931	Secreted binding protein	0.79	1.01
**Transport, secretion and efflux**				
C9Z5D4	SCAB_27411	Secreted oligopeptide-binding transport system protein	1.12	1.26
C9ZFJ5	SCAB_49311	Phosphate-binding protein PstS	0.83	0.82
C9YTX8	SCAB_67681	Bacterioferritin	0.84	0.79
C9ZA98	SCAB_29881	Binding protein	0.38	0.36
C9Z0C3	SCAB_8741	Solute-binding lipoprotein	0.32	0.21
C9Z0P2	SCAB_24881	Glutamate uptake system ATP-binding subunit	0.45	0.29
C9YWN6	SCAB_68891	ABC transporter ATP-binding subunit	0.29	0.35
C9YY97	SCAB_70061	ABC transporter ATP-binding subunit	0.14	0.25
C9YUK3	SCAB_19841	Lipoprotein	0.15	0.13
C9Z205	SCAB_24901	Glutamate uptake system	0.27	0.15
C9ZH25	SCAB_50031	Phosphate-specific transport system accessory protein PhoU	0.16	0.24
C9Z0I9	SCAB_24291	DNA topoisomerase	0.11	0.08
C9ZAS0	SCAB_61971	Binding-protein-dependent metal transporter ATP-binding subunit	0.24	0.41
C9YZP9	SCAB_70761	Secreted solute-binding protein	0.30	0.27

C9Z5D2	SCAB_27391	Oligopeptide ABC transporter component	0.28	0.24
C9Z3R1	SCAB_26231	Signal recognition particle protein	0.10	0.08
C9YZK9	SCAB_55371	Protein translocase subunit SecA	0.05	0.04
C9YX53	SCAB_83561	L-asparagine permease	0.09	0.09
C9Z206	SCAB_24911	Glutamate uptake system	0.09	0.09
C9YUG0	SCAB_5331	ATP-binding component of ABC transporter	0.09	0.22
C9YWN9	SCAB_68921	ABC transporter integral membrane subunit	0.18	0.11
C9YTT3	SCAB_67221	Integral membrane efflux protein	0.05	0.06
C9Z7A7	SCAB_59521	Mechanosensitive ion channel	0.05	0.08
C9ZA99	SCAB_29891	Binding-protein-dependent transporter	0.25	0.28
C9YWN8	SCAB_68911	ABC transporter integral membrane subunit	0.08	0.05
C9Z8C6	SCAB_28551	ABC transporter ATP-binding subunit	0.09	0.09
C9ZBZ2	SCAB_31501	Peptide ABC transporter	0.11	0.10
C9Z307	SCAB_73301	Glycerol uptake facilitator protein	0.22	0.13
C9YW00	SCAB_21251	RNA helicase	0.04	0.03
C9Z5D1	SCAB_27381	Oligopeptide ABC transporter	0.15	0.16
C9ZD96	SCAB_31521	BldKC-like oligopeptide ABC transporter subunit	0.08	0.11
C9ZE01	SCAB_64271	Efflux transporter	0.04	0.05
C9ZD98	SCAB_31541	BldKA-like ABC transport system integral membrane protein	0.13	0.05

C9YT14	SCAB_19051	Probable solute-binding lipoprotein	0.44	0.37
C9Z646	SCAB_74921	Protein-export membrane protein SecF	0.08	0.09
C9ZD95	SCAB_31511	BldKD oligopeptide ABC transporter subunit	0.36	0.24
C9Z000	SCAB_84391	Cobalt transport protein CbiN	0.23	0.20
C9ZD97	SCAB_31531	BldKB-like transport system extracellular solute-binding protein	0.39	0.42
C9Z2T0	SCAB_57661	Secreted solute-binding protein	0.66	0.56
C9YUG2	SCAB_5351	Substrate-binding component of ABC transporter	0.66	0.73
C9YVX8	SCAB_21021	Secreted solute-binding protein	0.79	0.87
**Energy production and conversion**				
C9YTR7	SCAB_67061	Dihydrolipoyl dehydrogenase	2.74	2.35
C9ZGW6	SCAB_34651	Oxidoreductase	0.62	0.66
C9YTC3	SCAB_35291	Succinate dehydrogenase flavoprotein subunit	0.45	0.50
C9Z8F0	SCAB_28781	ATP synthase subunit alpha	1.70	1.64
C9Z8F1	SCAB_28791	ATP synthase subunit delta	0.84	0.71
C9YTU0	SCAB_67291	Cytochrome c oxidase subunit II	0.66	0.50
C9YTR4	SCAB_67031	Pyruvate dehydrogenase E1 component	0.11	0.16
C9Z6Y2	SCAB_28231	Acetate kinase	0.28	0.19
C9YWX6	SCAB_82781	Hydrogenase	0.31	0.41
C9ZA89	SCAB_29781	2-oxoglutarate dehydrogenase	0.03	0.08

C9Z0U9	SCAB_39831	Inorganic pyrophosphatase	0.37	0.39
C9Z476	SCAB_58021	Acetyl/propionyl CoA carboxylase alpha subunit	0.15	0.17
C9Z7Q3	SCAB_75771	Acyl-CoA dehydrogenase	0.29	0.18
C9YTC4	SCAB_35301	Succinate dehydrogenase iron-sulfur subunit	0.34	0.29
C9YTU1	SCAB_67301	Cytochrome c oxidase subunit I	0.13	0.14
C9Z8V4	SCAB_45161	E2 branched-chain alpha keto acid dehydrogenase system	0.26	0.25
C9YYR1	SCAB_7801	Oxidoreducatse	0.10	0.07
C9ZAG4	SCAB_45351	Aldehyde dehydrogenase	0.20	0.22
C9Z6J6	SCAB_12211	Isocitrate dehydrogenase	0.16	0.21
C9YXW4	SCAB_38111	Oxidoreductase	0.20	0.14
C9Z306	SCAB_73291	Glycerol kinase	0.27	0.16
C9YVY6	SCAB_21101	Aconitate hydratase	0.18	0.25
C9YWN7	SCAB_68901	ABC transporter ATP-binding subunit	0.41	0.36
C9Z8F2	SCAB_28801	ATP synthase subunit b	1.35	1.35
C9YXQ1	SCAB_37431	NADH dehydrogenase subunit NuoM2	0.05	0.06
C9Z8F4	SCAB_28821	ATP synthase subunit a	0.24	0.22
C9YZL4	SCAB_55421	NAD-glutamate dehydrogenase	0.02	0.04
C9YTC2	SCAB_35281	Succinate dehydrogenase membrane subunit	0.11	0.11
C9Z5T2	SCAB_58931	Methylmalonic acid semialdehyde dehydrogenase	0.10	0.09

C9ZEP5	SCAB_16961	Oxidoreductase	0.10	0.12
C9Z8V5	SCAB_45171	E1-beta branched-chain alpha-keto-acid dehydrogenase system	0.19	0.20
C9ZE04	SCAB_64301	Pyruvate dehydrogenase E1 component	0.04	0.05
C9ZA96	SCAB_29851	Alcohol dehydrogenase	0.10	0.09
C9YTU7	SCAB_67361	Ubiquinol-cytochrome c reductase iron-sulfur subunit	0.09	0.16
C9ZFY0	SCAB_79811	Aldehyde dehydrogenase	0.06	0.06
C9ZBX1	SCAB_31271	Ferredoxin	0.67	0.42
C9ZF68	SCAB_33551	Fumarate hydratase class I	0.07	0.05
C9Z8V6	SCAB_45181	E1-alpha branched-chain alpha keto acid dehydrogenase system	0.08	0.16
C9YTM7	SCAB_51341	Citrate synthase	0.15	0.14
C9YUX5	SCAB_36201	IMP dehydrogenase/ GMP reductase	0.51	0.64
C9YTR6	SCAB_67051	Dihydrolipoyllysine-residue succinyltransferase	0.38	0.38
C9Z8E8	SCAB_28761	ATP synthase subunit beta	1.54	1.77
C9YTG2	SCAB_35681	Malate dehydrogenase	1.90	1.59
C9Z8E9	SCAB_28771	ATP synthase gamma chain	1.15	1.18
**General function prediction only**				
C9ZH64	SCAB_50441	60 Chaperonin	0.64	0.86
C9ZD54	SCAB_16471	DNA-binding protein	1.00	0.96
C9Z5G9	SCAB_42541	Chaperone protein DnaK	0.37	0.36

C9Z0R6	SCAB_39491	Clp-family ATP-binding protease	0.25	0.23
C9Z0U4	SCAB_39781	ATP-dependent zinc metalloprotease FtsH	0.22	0.27
C9ZCL9	SCAB_78431	Secreted tripeptidylaminopeptidase	0.28	0.25
C9YZC0	SCAB_39031	Delta-aminolevulinic acid dehydratase	0.35	0.35
C9YVQ4	SCAB_6071	Histidine kinase	0.34	0.22
C9Z2T5	SCAB_72541	Homogentisate 1,2-dioxygenase	0.21	0.24
C9Z3Q0	SCAB_26121	Signal peptidase	0.33	0.34
C9Z5E5	SCAB_42291	Chaperone protein ClpB	0.12	0.10
C9YUY5	SCAB_36301	10 Chaperonin	0.91	0.91
C9Z7F0	SCAB_59941	ATP-dependent Clp protease proteolytic subunit	0.30	0.34
C9ZAJ2	SCAB_45651	Peptidyl-prolyl cis-trans isomerase	0.34	0.39
C9Z0V9	SCAB_39931	Aldehyde dehydrogenase	0.17	0.12
C9ZGZ0	SCAB_49661	Uncharacterized protein	0.65	0.46
C9YXV0	SCAB_37971	NuoM, NADH dehydrogenase subunit	0.13	0.13
C9Z4D5	SCAB_73521	Peptidyl-prolyl cis-trans isomerase	0.26	0.15
C9Z474	SCAB_58001	Acyl-CoA dehydrogenase	0.20	0.18
C9Z589	SCAB_26951	3-isopropylmalate dehydrogenase	0.17	0.18
C9Z9C8	SCAB_76271	Uncharacterized protein	0.24	0.29
C9Z0Y6	SCAB_55671	Serine/threonine protein kinase	0.08	0.07

C9YXC1	SCAB_7101	Alcohol dehydrogenase class III	0.15	0.09
C9YUI1	SCAB_5561	Integral membrane protein	0.22	0.24
C9Z8W9	SCAB_45311	E1-alpha branched-chain alpha keto acid dehydrogenase	0.15	0.20
C9Z7I2	SCAB_60251	Peptidase	0.18	0.21
C9Z1U3	SCAB_9361	Prolyl aminopeptidase	0.08	0.08
C9Z210	SCAB_24951	Protein RecA	0.11	0.28
C9Z1F7	SCAB_72331	Hydrolase	0.15	0.19
C9ZAH6	SCAB_45481	Secreted protein	0.16	0.18
C9Z475	SCAB_58011	Hydroxymethylglutaryl-CoA lyase	0.32	0.29
C9YZ81	SCAB_38631	Cytochrome assembly protein	0.08	0.11
C9YYG1	SCAB_83671	Esterase	0.07	0.10
C9YZK1	SCAB_55281	Two-component system response regulator	0.14	0.22
C9ZGF2	SCAB_17921	Oxidoreductase	0.15	0.19
C9Z110	SCAB_55941	DNA-binding protein HU1/hs1	0.45	0.65
C9ZAC0	SCAB_30091	Anti-sigma factor	0.20	0.20
C9Z421	SCAB_42041	Cytochrome P-450 hydroxylase	0.07	0.06
C9Z6A5	SCAB_88391	2-hydroxyhepta-2,4-diene-1,7-dioate isomerase	0.10	0.06
C9Z1Q3	SCAB_85931	Regulatory protein	0.09	0.06
C9ZAL7	SCAB_45921	Thioredoxin	0.21	0.17

C9ZAI3	SCAB_45561	Penicillin-binding kinase	0.04	0.07
C9Z7F1	SCAB_59951	ATP-dependent Clp protease proteolytic subunit	0.20	0.26
C9ZC20	SCAB_46561	Serine--tRNA ligase	0.05	0.09
C9Z477	SCAB_58031	Acetyl/propionyl CoA carboxylase, beta subunit	0.21	0.24
C9ZD20	SCAB_16121	Serine/threonine-protein kinase	0.06	0.05
C9YWG0	SCAB_53111	Secreted penicillin acylase	0.35	0.27
C9ZDR6	SCAB_47861	Phosphoribosylformylglycinamidine cyclo-ligase	0.50	0.46
C9YUY4	SCAB_36291	60 Chaperonin	0.51	0.50
**Unknown function**				
C9Z4C0	SCAB_58481		0.74	0.79
C9YVE9	SCAB_68241		0.41	0.47
C9Z3A4	SCAB_86841		1.21	1.32
C9YZX4	SCAB_71541		0.56	0.46
C9ZBU0	SCAB_30961		0.46	0.57
C9ZB66	SCAB_77921		0.22	0.22
C9ZBP7	USCAB_30501		0.11	0.14
C9ZH08	SCAB_49851		0.57	0.64
C9Z064	SCAB_85031		0.72	0.67
C9YWC0	SCAB_37371		0.72	0.58

C9YUE9	SCAB_5221	0.38	0.40
C9YTS9	SCAB_67181	0.23	0.30
C9Z6S2	SCAB_27611	0.13	0.13
C9ZHB0	SCAB_66321	0.30	0.26
C9Z4A2	SCAB_58301	0.29	0.21
C9ZHE9	SCAB_66721	0.54	0.42
C9YWC3	SCAB_37401	0.53	0.27
C9Z4V1	SCAB_87931	0.13	0.35
C9ZGX1	SCAB_34721	0.30	0.20
C9YWN2	SCAB_68851	0.19	0.22
C9Z7H6	SCAB_60191	0.07	0.08
C9YVV6	SCAB_20801	0.23	0.16
C9Z4H0	SCAB_73871	0.28	0.13
C9ZCE7	SCAB_63371	0.14	0.15
C9YZ82	SCAB_38641	0.08	0.09
C9ZAJ4	SCAB_45671	0.29	0.21
C9Z723	SCAB_43511	0.38	0.22
C9Z6P3	SCAB_12751	0.13	0.10
C9YUT3	SCAB_35761	0.20	0.15

C9ZEV2	SCAB_17551	0.25	0.31
C9ZFR1	SCAB_65491	0.14	0.18
C9Z436	SCAB_42191	0.16	0.14
C9YTZ7	SCAB_67871	0.30	0.23
C9Z0I7	SCAB_24261	0.06	0.10
C9Z481	SCAB_58071	0.14	0.14
C9Z7E2	SCAB_59881	0.11	0.07
C9Z491	SCAB_58181	0.11	0.14
C9YW84	SCAB_37001	0.14	0.13
C9YZJ9	SCAB_55261	0.10	0.06
C9YVH2	SCAB_68471	0.33	0.44
C9Z871	SCAB_13411	0.11	0.08
C9Z565	SCAB_26701	0.25	0.25
C9Z6Z9	SCAB_28401	0.13	0.15
C9ZHG7	SCAB_66901	0.12	0.14
C9Z8D0	SCAB_28591	0.21	0.25
C9ZHJ9	SCAB_80831	0.05	0.05
C9Z0I8	SCAB_24271	0.07	0.06
C9ZBQ8	SCAB_30621	0.13	0.13

C9YXY2	SCAB_53831	0.08	0.11
C9Z0R8	SCAB_39511	0.25	0.25
C9Z567	SCAB_26721	0.10	0.17
C9Z4Y9	SCAB_11251	0.38	0.31
C9ZBT8	SCAB_30941	0.25	0.44
C9ZAX3	SCAB_62551	0.18	0.27
C9ZAJ1	SCAB_45641	0.09	0.06
C9YTS8	SCAB_67171	0.10	0.10
C9ZBZ4	SCAB_46281	0.18	0.23
C9ZEW2	SCAB_17651	0.11	0.13

NSpC-C: normalized spectral count in CM (see Materials and Methods).
NSpC-F: normalized spectral count in CM supplemented with *trans*-ferulic (see Materials and Methods).
